# Phytoplankton Impact on Marine Cloud Microphysical Properties Over the Northeast Atlantic Ocean

**DOI:** 10.1029/2021JD036355

**Published:** 2022-05-24

**Authors:** Karam Mansour, Matteo Rinaldi, Jana Preißler, Stefano Decesari, Jurgita Ovadnevaite, Darius Ceburnis, Marco Paglione, Maria C. Facchini, Colin O'Dowd

**Affiliations:** ^1^ Italian National Research Council ‐ Institute of Atmospheric Sciences and Climate (CNR‐ISAC) Bologna Italy; ^2^ Oceanography Department, Faculty of Science Alexandria University Alexandria Egypt; ^3^ Leosphere Saclay France; ^4^ School of Physics Ryan Institute's Centre for Climate and Air Pollution Studies National University of Ireland Galway Galway Ireland

**Keywords:** phytoplankton, cloud microphysics, cloud optical properties, marine aerosol, North Atlantic, SYRSOC

## Abstract

The current understanding of the impact of natural cloud condensation nuclei (CCN) variability on cloud properties in marine air is low, thus contributing to climate prediction uncertainty. By analyzing cloud remote sensing observations (2009–2015) at Mace Head (west coast of Ireland), we show the oceanic biota impact on the microphysical properties of stratiform clouds over the Northeast Atlantic Ocean. During spring to summer (seasons of enhanced oceanic biological activity), clouds typically host a higher number of smaller droplets resulting from increased aerosol number concentration in the CCN relevant‐size range. The induced increase in cloud droplet number concentration (+100%) and decrease in their radius (−14%) are comparable in magnitude to that generated by the advection of anthropogenically influenced air masses over the background marine boundary layer. Cloud water content and albedo respond to marine CCN perturbations with positive adjustments, making clouds brighter as the number of droplets increases. Cloud susceptibility to marine aerosols overlaps with a large variability of cloud macrophysical and optical properties primarily affected by the meteorological conditions. The above findings suggest the existence of a potential feedback mechanism between marine biota and the marine cloud‐climate system.

## Key Points

- Ground‐based observations show variations in cloud droplet number (+100%) and their radius (−14%) associated with high phytoplankton activity
- The biota‐induced variations in stratiform cloud microphysics are comparable in magnitude to those generated by anthropogenic inputs
- Cloud optical properties mainly respond to meteorology, however, the increase in summer droplet number contributes to enhanced albedo

## 1 Introduction

Stratiform clouds mostly originate over the oceans and have a profound impact on the Earth's radiation budget. Therefore, understanding the causes of the variability in their radiative effects is necessary for a better comprehension of the Earth's energy budget and climate forecast. The future effort to reduce the impact of greenhouse gases and their associated global warming potential puts the emphasis on natural sources (Boucher et al., 2013; Satheesh & Moorthy, 2005) and processes controlling marine stratiform clouds.

The radiative impact and brightness of clouds strongly depend on their microphysical properties (Falkowski et al., 1992) such as liquid water content (LWC), cloud droplet effective radius (R_eff_), and cloud droplet number concentration (CDNC). At constant LWC, the increase of aerosol number and cloud condensation nuclei (CCN) atmospheric concentrations results in higher CDNC while the R_eff_ diminishes. Therefore, clouds are optically brighter as more solar radiation is scattered upward and less is transmitted through. This is known as the first aerosol‐cloud indirect effect (Twomey effect; Twomey [1974]) and represents the main factor regulating the instantaneous radiative forcing from aerosol‐cloud interaction (RF_aci_). The induced changes of cloud microphysics can affect the onset of precipitation, cloud water path, and cloud cover according to a range of adjustment mechanisms which may either amplify or dampen the ACI radiative effect (Albrecht, 1989; Rosenfeld et al., 2019) and that may be as important as the instantaneous effects. Such mechanisms have been a subject of intensive research in the case of marine stratus and stratocumulus clouds which represent one of the most common cloud types (in terms of relative cloud fraction) over the Earth. In particular, the oceanic regions covered by stratocumulus clouds exhibit a higher sensitivity of cloud albedo to aerosol perturbations (Gryspeerdt et al., 2019). Aerosol‐cloud interactions are challenging to investigate because the cloud response to changes in aerosol concentration and composition is intertwined with the response to variations in the meteorological conditions (Loeb & Schuster, 2008). Therefore, it is difficult to separate aerosol effects from meteorological impacts (Sato & Suzuki, 2019; Stevens & Feingold, 2009). In the marine boundary layer (MBL), cloud cover is generally associated with lower troposphere stability (LTS; Janssen et al. [2011]; Wood & Bretherton [2006]), a measure of convection strength. A larger cloud fraction is generally associated with higher LTS (Andersen et al., 2017; Klein & Hartmann, 1993). The transport and mixing of aerosols from the surface to the cloud base are essential since they influence the proportion of particles that activate into cloud droplets (Janssen et al., 2011). Excluding the meteorological impact, Rosenfeld et al. (2019) concluded that aerosol‐driven droplet concentrations account for 75% of the radiative cooling effect variability, through their effects on the coverage of low‐level ocean clouds.

Marine biota is a major source of biogenic aerosols in the atmosphere, through both primary and secondary aerosol formation pathways. The present limited knowledge of such natural emissions contributes to the high uncertainties in aerosol‐cloud forcing estimates (Carslaw et al., 2013; Rap et al., 2013). Most of the research done so far in this regard is focused on the Southern Ocean, which is considered a unique environment because of its remoteness. In such conditions, natural climate interactions can be more easily investigated, without interference from anthropogenic sources (McCoy et al., 2015). Cloud droplets have been often reported to be more numerous and smaller in size over oceanic regions characterized by high biological activity (HBA) than over low biological activity (LBA) regions (McCoy et al., 2015; Meskhidze & Nenes, 2006; Sorooshian & Duong, 2010; Sorooshian et al., 2009). Laboratory experiments and field observations have elucidated that the increase in CCN concentration is linked to phytoplankton abundance (Collins et al., 2016; Fossum et al., 2018, 2020; Mayer et al., 2020; Sellegri et al., 2021), where the effect of human activities appears to be minimal (Falkowski et al., 1992). Contrasting conclusions were presented by Miller and Yuter (2008) who did not always find a correlation between oceanic biological activity and R_eff_, questioning the existence of a causal link between secondary aerosols and marine shallow cloud properties. This ambiguity may reflect the complexity of studying marine biogenic aerosol‐cloud interactions, while at the same time some studies may have been biased by dominant continental aerosols as noted by Meskhidze and Nenes (2010).

In this work, we study the CCN variability and ocean biota impacts on cloud properties in unperturbed conditions over the Northeast Atlantic (NEA) Ocean. The NEA Ocean exhibits a variety of seasonal phytoplankton biomass patterns, with a distinct bloom beginning mostly in spring and continuing through the summer (Friedland et al., 2016; Lacour et al., 2015). Such biological activity has been proven to impact the chemical composition, number concentration, and size distribution of marine aerosols (Ault et al., 2013; Facchini et al., 2008; O'Dowd et al., 2004; Prather et al., 2013), which would eventually change cloud characteristics (Charlson et al., 1987). In this region, a few studies have reported a link between oceanic biological activity and cloud properties. For instance, an increase of about 15%–100% in CDNC was attributed to the impact of marine organic aerosols by using a mono‐dimensional cloud droplet model (O'Dowd et al., 2004). Sea spray enriched with primary organics enhances the CCN activation efficiency leading to high CDNC concentration during less than 6 hr of cloud observations (Ovadnevaite et al., 2011). Such influence was evident in the summer when biological activity is maximized (Gantt et al., 2012). On the contrary, some studies lessened the relative importance of the sea‐spray organic fraction in determining its CCN properties (Hendrickson et al., 2021), suggesting that non‐sea‐salt sulfate may be the strongest link between marine biology and CCN (Quinn et al., 2019; Saliba et al., 2020; Sanchez et al., 2018).

In addition to the complexity of cloud properties adjustment and feedback processes affecting the sign and magnitude of ACI radiative effects, there is also a methodological problem in the assessment of such effects from the observational point of view (Gryspeerdt et al., 2019), as most common remote sensing techniques cannot provide full vertical profiling of all relevant properties for both clouds and aerosols. Cloud observations are usually carried out at the cloud top, with the microphysics of the main entrainment zone of aerosol‐rich air (at cloud base) being inaccessible. In this study, we employ a combination of ground‐based aerosol in situ and cloud remote sensing observations to overcome the retrieval limitations of satellite‐based techniques while providing much wider temporal coverage (ca. 6 years of observations) with respect to in situ measurements available from aircraft missions (Sinclair et al., 2020).

The Mace Head (MHD) global atmosphere watch research station (53.33°N, 09.90°W; 21 m a.s.l), located about 80 m from the waterline, on the west coast of Ireland, is exposed to the North Atlantic Ocean through the westerly sector. Stratiform clouds at MHD have a prevailing marine origin. Numerous studies of in situ observations of clouds have been done through the MHD remote sensing unit, including the development of the SYnergistic Remote Sensing Of Clouds (SYRSOC) algorithm (Martucci & O'Dowd, 2011). This retrieval algorithm was further extensively used to combine in situ ground aerosol and cloud measurements (Martucci et al., 2013; Ovadnevaite et al., 2011). The marine and continental stratiform clouds were characterized by Preiβler et al. (2016) who found that a lower CDNC with broader R_eff_ during clean marine conditions than in continental cases, in line with the comprehensive in situ database of stratus cloud (Miles et al., 2000).

In this study, we assess the relationship between oceanic biological activity and cloud properties in background marine conditions, over the NEA Ocean, by exploring a unique dataset of multi‐year cloud observations (from February 2009 to January 2015) measured by ground‐based remote sensing instruments at MHD and retrieved by SYRSOC method. The data include cloud microphysical (CDNC, R_eff_, LWC), cloud macrophysical (cloud base height [H_base_], cloud top height [H_top_], and cloud thickness [H_thick_]), and cloud optical properties (albedo and cloud optical depth [COD]). Cloud observations were analyzed together with satellite ocean color data (sea surface chlorophyll‐a concentrations (CHL) as a proxy of phytoplankton activity), air mass back‐trajectories and in situ aerosol particle number concentration (N_a_) data in the CCN relevant size range between 40 and 300 nm in diameter (Quinn & Bates, 2011). Finally, using ECMWF‐ERA5 reanalysis atmospheric data, we analyze the main meteorological drivers for stratiform cloud formation in this region. Based on that, we discriminate the effect of meteorology on cloud properties from that of ACI processes.

## 2 Materials and Methods

### 2.1 Ground‐Based Remote Sensing Measurements

The ground‐based remote sensing (GBRS) division at MHD, on the west coast of Ireland, has been a Cloudnet station since 2009 (Illingworth et al., 2007) and comprises cloud radar, ceilometer, and microwave radiometer (MWR), and since 2015 a Doppler wind lidar. GBRS provides continuous monitoring of the atmosphere at one location, with high vertical and temporal resolutions. This provides useful detailed insights into cloud processes, which can be a powerful tool for the detection and quantification of cloud indirect effects (Feingold et al., 2003).

The radar is a MIRA36, a 35.5 GHz Ka‐band Doppler cloud radar (Melchionna et al., 2008) that measures in‐cloud reflectivity, and linear depolarization ratio, and vertical speed of cloud droplets. The radar was also used to track the top level of clouds. Further description of the calibration offset of the radar reflectivity can be found in Preiβler et al. (2016).

The ceilometer is a CHM15k measuring 1,064 nm (Heese et al., 2010; Martucci et al., 2010). It detects photons backscattered from atmospheric targets such as cloud droplets or aerosol particles. Based on the optical depth of the atmosphere, it can detect aerosol particles, as well as clouds up to a certain penetration point. The ceilometer was used to detect the cloud base altitude.

The MWR is a multichannel microwave profiler RPG‐HATPRO (Crewell & Lohnert, 2003; Lohnert & Crewell, 2003; Lohnert et al., 2009) measuring near water vapor and oxygen absorption lines (Martucci & O'Dowd, 2011).

The SYRSOC retrieval method uses GBRS data from the three abovementioned instruments as a primary input. It allows for vertically resolved determination of cloud properties from the ground. Using the SYRSOC algorithm (Martucci & O'Dowd, 2011; Preiβler et al., 2016), the microphysical, macrophysical, and optical properties are retrieved. Homogeneous sections of less than 1‐hr duration have been selected of all non‐precipitating single‐layer water clouds. The microphysical properties of each cloud case are represented by a 2D matrix with time as the *x*‐axis and height as the *y*‐axis. The vertical and temporal resolutions are 15 m and 10 s.

Starting from the 6 years (February 2009 to January 2015) of SYRSOC dataset cloud observations (Preiβler, 2016), the air mass back‐trajectories were calculated using the Hybrid Single‐Particle Lagrangian Integrated Trajectory (HYSPLIT4) model, a transport and dispersion model developed by the National Oceanic and Atmospheric Administration (NOAA), Air Resources Laboratory (ARL; Rolph et al. [2017]; Stein et al. [2015]) to define the marine source areas, coming from the Atlantic Ocean. Based on back‐trajectories (Figure S1 in Supporting Information S1), a total of 52 clean marine stratiform clouds were classified as a representative of the North Atlantic Ocean background and used in this study. The cloud cases are distributed over 47 days. The ground level average equivalent black carbon measured in situ by a multi‐angle absorption photometer (O'Dowd et al., 2014) associated with the considered 52 cases is 8.8 ± 5.2 ng/m^3^, proving negligible anthropogenic contribution.

The cloud microphysics retrievals from SYRSOC were compared with in situ aircraft measurements from a 2‐day field experiment conducted at MHD (Martucci et al., 2013), showing a very good agreement in the SYRSOC full profiles. Nevertheless, we compared the SYRSOC results of the selected cases with the Moderate‐Resolution Imaging Spectroradiometer (MODIS) level‐2 cloud product from both Terra (MOD06) and Aqua (MYD06) platforms (Platnick et al., 2017). The comparison is explained in the supplementary materials (see Text S1 in Supporting Information S1) and shown in Figure S2 in Supporting Information S1. The R_eff_ by SYRSOC is slightly lower than that by MODIS, consistently with the whole dataset (Preiβler et al., 2016). CDNC is more scattered, consistently with the fact that it is still a very uncertain parameter (Grosvenor et al., 2018), nevertheless, the data fall overall around the 1:1 line.

### 2.2 In Situ Measurements

The submicron aerosol size distributions were measured by a Scanning Mobility Particle Sizer (0.02 < particle diameter (Dp) < 0.5 μm), where aerosols are neutralized (aerosol neutralizer: Kr‐85, TSI Model 3077) and size‐discriminated based on their mobility diameter (differential mobility analyzer: TSI Model 3071) and counted by a condensation particle counter (TSI Model 3010). Aerosol sizing instruments were located downstream of Nafion driers (relative humidity <20%; Ovadnevaite et al. [2017]), and thus, size distributions refer to the aerosol dry diameter (Sanchez et al., 2017). We have selected the particle number concentration of 40 < Dp < 300 nm for this study since this diameter range is large enough to take up water vapor and serve as nuclei for cloud droplet formation in MBL (Quinn & Bates, 2011).

### 2.3 Satellite Ocean Color Data

The present study is based on the best estimates of “Level‐4 Cloud Free” satellite ocean‐color data products which are the result of merging Sea‐viewing Wide Field of View (SeaWiFS), Moderate Resolution Imaging Spectroradiometer (MODIS‐Aqua), MEdium Resolution Imaging Spectrometer (MERIS), Visible and Infrared Imager/Radiometer Suite (VIIRS) and Ocean and Land Color Instrument‐Sentinel 3A (OLCI‐S3A) sensors. The sea surface CHL obtained from the EU Copernicus Marine Environment Monitoring Service (CMEMS) was used as the reference surrogate for tracing the evaluation of marine biological activity (Rinaldi et al., 2013; Yoon et al., 2007) because it is the most widely available and validated ocean color parameter. CHL is commonly used as a proxy of phytoplankton activity in seawater (Behrenfeld et al., 2016; Huot et al., 2007); the more phytoplankton present, the greener the water and the higher CHL. For the NEA Ocean domain (40°–46°N and 46°–0°W), CHL fields were extracted from a global product, available at 1/24° (about 4 km) spatial resolution and daily time‐resolution. The analyzed time series covers the period from 2009 to 2015.

### 2.4 Meteorological Data

The European Centre for Medium‐Range Weather Forecasts (ECMWF) fifth generation reanalysis ERA5 (C3S, 2017) data provides estimates for the hourly state of the atmosphere, worldwide, with spatial resolution of 0.25° × 0.25° at the surface and different pressure levels. The ERA5 data are available by the ESA Ocean Color‐Climate Change Initiative (ESA OC‐CCI) team and the Copernicus climate change service (C3S). From the global domain, we extracted the following meteorological parameters in order to evaluate the relationship between cloud properties and the main meteorological variables in comparison with the importance of the oceanic biological activity.

The air temperature at 2m above sea level (T) and sea surface temperature (SST) was chosen as representative of thermal heating and the relative humidity (RH) was chosen as representative of water vapor abundance in the atmosphere. To represent the atmospheric thermodynamic and dynamic conditions, the lower tropospheric stability (LTS) parameter and the pressure vertical velocity (PVV) were utilized, respectively.

The LTS, an indicator for the air convection, is calculated as the potential temperature difference between 1,000 and 850 hPa (Fuchs et al., 2017; Painemal, 2018), which reflects thermal (in)stability in the sub‐cloud layer: the higher the LTS, the more unstable the atmosphere. For the 52 studied marine cloud cases, the LTS ranges from −1.8 to 10.9°C. Positive values of LTS account for 88% of the cases, indicating a dominant thermal instability for the selected cloud cases. The pressure vertical velocity (PVV; Pa s^−1^) is the speed at which the air travels up or down. Negative values of PVV imply upward motion (ascent areas), while positive values represent downward motion (subsidence).

### 2.5 Spatio‐Temporal Correlation Analysis

To investigate the relationship between marine cloud properties and oceanic biological activity (represented by CHL), we implemented the spatio‐temporal correlation analysis approach (Mansour, Decesari, Bellacicco, et al., 2020; Mansour, Decesari, Facchini, et al., 2020). The approach is based on evaluating, by standard least squares regression, the correlation coefficients between the GBRS cloud parameters (the average of each variable in the 2D cloud profile; vertically‐ cloud thickness and horizontally‐ measurement time), measured at MHD, and sea surface CHL, at each grid point of the NEA domain. The justifications and full details of the approach can be found in the above‐cited papers. Basically, we tested the effect of the time‐lag on the “cloud properties versus CHL” correlations to compensate for the different phases of the correlated variables. Indeed, surface CHL tracks the growing phase of algal blooms, while the release of phytoplankton exudates, involved in the production of biogenic marine aerosols, occurs mainly at a later stage, during the senescence/demise phase (Miyazaki et al., 2020; Rinaldi et al., 2013). Starting from simultaneous time series, corresponding to “Lag = 0”, we shifted CHL backward day by day to calculate the correlation coefficient between the two considered time series. Such a process is conducted for each pixel of the studied domain, to obtain the “Lag = n days” correlation maps (Figures S3‐S5 in Supporting Information S1) where “n” is the number of days by which the CHL time‐series is shifted back.

### 2.6 Aerosol‐Cloud Interaction Index

To examine the microphysical response of cloud to aerosol loading, the ACI indices for both R_eff_ and CDNC were calculated as:

$${\text{ACI}}_{r}=-\frac{d\;\ln\left(R_{\text{eff}}\right)}{d\;\ln\left(N_{a}\right)}$$

$${\text{ACI}}_{n}=\frac{1}{3}\frac{d\;\ln\left(\text{CDNC}\right)}{d\;\ln\left(N_{a}\right)}$$

Both ACI_r_ and ACI_n_ (Koike et al., 2019; McComiskey & Feingold, 2012; Zhao et al., 2012) emphasize the relative change in the mean layer of R_eff_ and CDNC to the relative change in the aerosol loading and must be calculated at a constant LWC. The ACI values range from 0 to 0.33 where the lower boundary implies no change in cloud microphysical properties with aerosol variation and the upper boundary suggests a linear relationship.

The ACI must be calculated and compared at constant LWC, due to the dependence of R_eff_ and CDNC on LWC. To match the time‐resolution of aerosol number concentration, the cloud data were averaged vertically from base to top. Then, we used three LWC bins classified as Low, Medium, and High, using the one‐third and two‐third percentiles as threshold values. The LWC bins are <0.187 (*n* = 97), 0.187–0.276 (*n* = 97), and >0.276 gm m^−3^ (*n* = 97).

## 3 Results

### 3.1 Cloud Properties in Relation to CHL Patterns

**Figure 1:**
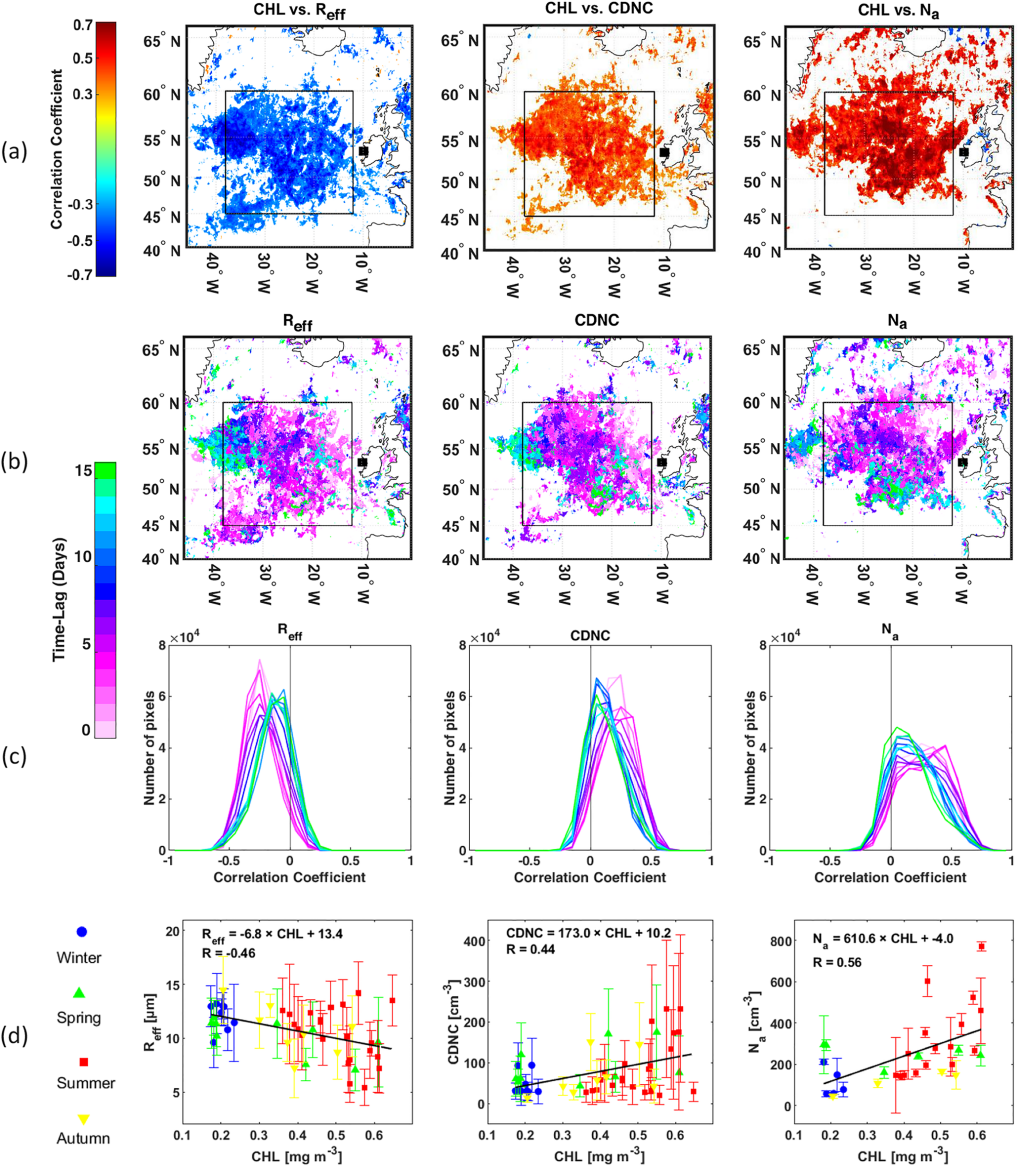
(a) Spatial distributions of the maximum correlation coefficient, within the time‐lags from 0 to 15 days, between chlorophyll‐a concentrations at each pixel of the Northeast Atlantic ocean and R_eff_, cloud droplet number concentration (CDNC) and N_a_ measured at Mace Head (MHD). (b) Spatial distribution of the time‐lags maximizes the correlation coefficient used in panel (a). Only significant correlation coefficients (*p < 0.05*) are presented. In (a) and (b) panels, the filled black square corresponds to the MHD station, the black box area comprises grid coordinates 45°−60°N and 12°−38°W that indicates a high correlation area, and the spatial resolution of the pixels is roughly 4 km. (c) Correlation coefficient frequency distributions in the identified box at different time‐lags from 0 to 15 days (d) Scatter plots between average CHL in the identified black box and average R_eff_, CDNC, and N_a_ for each cloud case; a 3‐day time‐lag, which maximizes the correlation, was considered. The significant (*p < 0.05*) correlation coefficients and the best fit line are reported. The vertical bars represent the standard deviation for each cloud case.

Following the spatiotemporal correlation analysis approach (Mansour, Decesari, Bellacicco, et al., 2020; Mansour, Decesari, Facchini, et al., 2020; O'Dowd et al., 2015; Rinaldi et al., 2013), we investigated the spatial distributions of the correlation coefficient between cloud properties observed at MHD and sea surface CHL at each grid point of the NEA domain, considering different time‐lags from 0 to 25 days. Among all the analyzed cloud metrics, only R_eff_ and CDNC demonstrated a robust and significant spatial correlation with CHL, which began to gradually diminish after time‐lag of 15 days. The resulting maps are presented in Figures S3 and S4 in Supporting Information S1. The synthetic maps in Figure 1a were created by selecting the maximum correlation obtained at each pixel within the range of time‐lags between 0 and 15 days. The spatial distributions of the time‐lags which maximize the correlation are shown in Figure 1b. In general, a systematic correlation was observed within a specific oceanic region of the NEA domain (45°−60°N and 12°−38°W: black box in Figure 1a), with correlations generally maximized considering a time‐lag between 0 and 5 days as indicated in Figure 1b. Overall, R_eff_ correlated negatively with CHL, while a positive correlation was observed for CDNC.

Importantly, N_a_ is positively correlated with CHL in the same identified oceanic region and presents a similar spatio‐temporal pattern of the correlation as CDNC and R_eff_ (Figure 1 and Figure S5 in Supporting Information S1). It follows that the observed correlation between cloud microphysical properties and CHL may indeed evidence a causal link between marine biological activity and cloud microphysical properties. This relation appears to be modulated by the influence that biological activity exerts on the atmospheric concentration of marine aerosol particles. Furthermore, we stress that the high‐correlation oceanic region evidenced in this study corresponds closely to the main source of biogenic marine aerosol observed at MHD documented in previous studies (Mansour, Decesari, Facchini, et al., 2020; Rinaldi et al., 2013), further supporting a cause‐effect link at the base of the observed correlations.

The correlation coefficient frequency distributions (Figure 1c), taken over the same box region, for time‐lags from 0 to 15 days (whereas the frequency distribution up to 25 days is shown in Figure S6 in Supporting Information S1) confirm the correlation between CHL and R_eff_ (CDNC and N_a_) is oriented toward negative (positive) values as the time‐lag is increased from 0 to 5 days. Furthermore, the percentages of the number of pixels with a negative (positive) significant correlation in that region for R_eff_ (CDNC and N_a_) demonstrate that the correlation begins to decline after a time lag of 15 days (Figure S7 in Supporting Information S1). In previous studies, a significant correlation could be observed between cloud‐relevant aerosol properties and CHL patterns, with a time‐lag between zero and up to 25 days (Mansour, Decesari, Facchini, et al., 2020; O'Dowd et al., 2015). Figure S8 in Supporting Information S1 complements this plot by looking at the correlation as a function of the time lag, considering the average CHL concentration within the high correlation region evidenced in Figure 1a, for all the tested cloud parameters.

Nevertheless, aware that the correlation analysis alone cannot unambiguously demonstrate a cause‐effect relationship between phytoplankton patterns and cloud properties, we applied the advanced three‐dimensional concentration weighted trajectory (3D‐CWT) model (Rinaldi et al., 2021) to the MHD aerosol and cloud data in order to identify the NEA ocean regions associated with higher N_a_/CDNC and lower R_eff_. The details of the analysis are presented in the Supporting Information S1 (Text S2). The resulting CWT maps (Figure S9 in Supporting Information S1) show a clear spatial consistency between sources of N_a_ and ocean regions associated with high‐CDNC/low‐R_eff_ clouds, which strengthens the above considerations on the aerosol influence on cloud microphysical properties. Furthermore, these same regions are clearly matching the high correlation region evidenced by the maps shown in Figure 1. To evidence this result, in Figure S10 in Supporting Information S1 we reported how many times a given pixel of the domain presented CWT ≤ median (CWT ≥ median) and negative (positive) significant correlation coefficient, in the case of R_eff_ (CDNC and N_a_), by considering the time‐lag from 0 to 15 days. This result clearly confirms that oceanic regions characterized by a significant correlation between aerosol and cloud parameters with surface CHL are also dominant sources of marine biogenic aerosol, characterized by the formation of clouds with higher CDNC and lower R_eff_.

The reported correlations (CHL vs. R_eff_, CDNC, and N_a_) are driven by the seasonal variability of the considered parameters, as shown in Figure 1d and Table S1 in Supporting Information S1 which evidence that seasons of enhanced sea surface CHL (spring and summer) are also characterized by low R_eff_ and by high N_a_ and CDNC. Typical LBA conditions, as observed during this study for the NEA Ocean, are characterized by surface CHL in the range of 0.13–0.40 mg m^−3^ (min & max over the box in Figure 1a) during the winter season (Dec‐Feb). These conditions are typically associated with R_eff_ and CDNC ranging between 9.6 and 13.2 μm and 16.8–86.3 cm^−3^, respectively. Conversely, the peak of biological productivity was represented by CHL between 0.33 and 0.93 mg m^−3^ in summer (Jun‐Aug). During this period, R_eff_ and CDNC ranged 5.4–14.2 μm and 15–213 cm^−3^. In conclusion, CDNC increased from 40 ± 26 to 81 ± 63 cm^−3^ [median 28 to 54], R_eff_ diminished from 12.1 ± 1.2 to 10.3 ± 2.5 μm [median 12.5 to 10.8] while the N_a_ ranged from 106 ± 62 cm^−3^ to 326 ± 186 [median 78 to 280] cm^−3^ going from winter to summer (Table S1 in Supporting Information S1). According to the nonparametric Mann‐Whitney U‐test for similarity of medians of two independent samples, the differences between winter and summer are statistically significant at *p* < 0.05 for CHL, N_a_, and R_eff_ whereas all of the reported variables are significantly different at *p* < 0.1.

For comparison purposes, Table S2 in Supporting Information S1 also presents a statistical summary of the cloud microphysical parameters obtained by selecting the 24 most polluted cloud cases from the extended dataset (Preiβler et al., 2016). These cases represented the advection of air masses from Europe over the NEA Ocean. The mean CDNC of the selected polluted cases was 209 ± 146 cm^−3^, corresponding to an average R_eff_ of 8.0 ± 2.4 μm. The average equivalent black carbon concentration for the polluted cases was equal to 231 ± 219 ng m^−3^, an order of magnitude higher than the marine background level of less than 15 ng m^−3^ (see Discussion for further comparison). It is worth highlighting that the seasonality of polluted cases has an opposite effect of marine cases (i.e., higher CDNC and lower R_eff_ in winter), as indicated in Figure S11 in Supporting Information S1.

### 3.2 Aerosol Particle Number Concentration and Cloud Microphysical Properties

**Figure 2:**
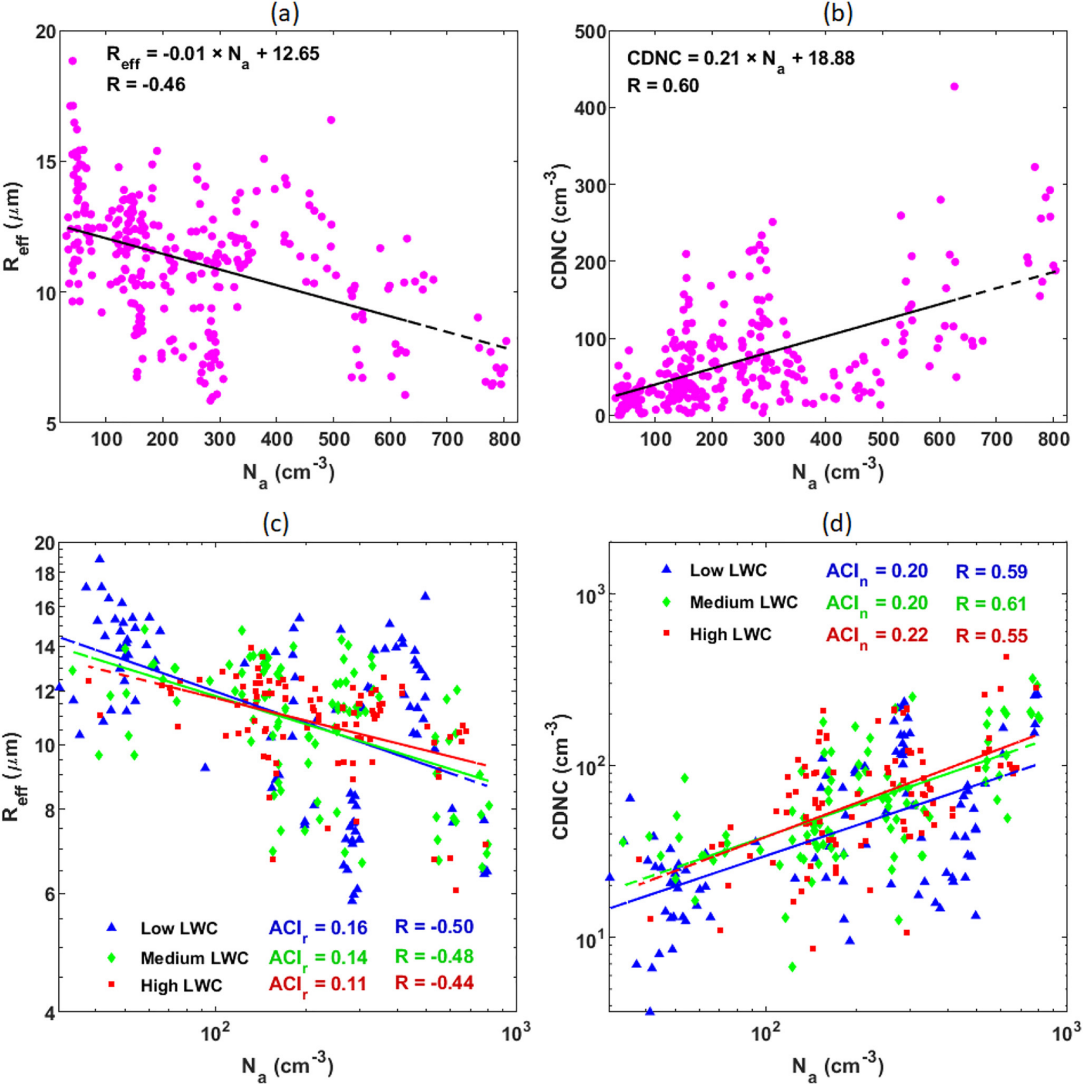
(a) Scatter plot between N_a_ and R_eff_. (b) Scatter plot between N_a_ and cloud droplet number concentration (CDNC). (c) and (d) aerosol‐cloud interaction (ACI)_r_ derived from R_eff_ to N_a_ and ACI_n_ derived from CDNC to N_a_ at three liquid water content (LWC) bins classified as LWC < third percentile (blue), one‐third percentile < LWC < two‐third percentile (green) and LWC > two‐third percentile (red); see Section 2.6 for the motivation of selecting homogeneous LWC classes in calculating ACI. All regression lines are significant (*p* < 0.05) whereas the differences between the slopes at different LWC classes are non‐significant (*p* < 0.05).

Looking at the N_a_‐R_eff_ and N_a_‐CDNC linear relationships reported in Figures 2a and 2b respectively, R_eff_ decreases with increasing N_a_ concentration while CDNC naturally increases with increasing N_a_, which is consistent with the well‐established Twomey effect (Twomey, 1974). Despite an apparent data scattering, likely arising from comparing ground measurements with ground remote sensing retrievals as well as from differences in other controlling factors such as updraft velocity, aerosol size distributions, and aerosol composition, the two regressions are statistically significant (*p* < 0.05). From the linear regression analysis, it can be inferred that the variations in aerosol concentration describe up to 21% and 36% of the R_eff_ and CDNC variance, respectively.

We quantified the cloud microphysical response to aerosol loading by using the quantitative ACI index (the slope of the relationship between aerosol loading and cloud parameter in log‐log space). At constant LWC, the ACI index evaluates the susceptibility of R_eff_ and CDNC to changes in aerosol number concentration, by varying between 0 (no effect at all) to 0.33 (linear response). The N_a_‐R_eff_ and N_a_‐CDNC relationships, grouped for homogeneous LWC classes, are presented in Figures 2c and 2d, respectively. Both ACI_r_ and ACI_n_ from the three LWC bins are positive, implying that clouds are sensitive to aerosol number concentration regardless of the amount of liquid water in the atmosphere (the differences between slopes are non‐significant at *p < 0.05*). The range of ACI_r_ values observed (0.11–0.16) is consistent with previous findings using ground‐based measurements. For instance, ACI_r_ values ranged from 0.04 to 0.17 (Kim et al., 2008), using a triennial analysis (1999‐2001). The ACI_r_ values between 0.02 and 0.19 were derived from an intensive operation period during May 2003 at a continental site of the United States (Feingold et al., 2003) and around 0.10–0.19 based on in situ aircraft observations in September 2015 over Hebei, China (Zhao et al., 2018). By considering in situ ground‐based remote sensing for stratiform clouds, the ACI_r_ values ranged from 0.05 to 0.16 on the California coast (McComiskey et al., 2009) and from 0.13 to 0.19 in the Arctic regions (Garrett et al., 2004). In addition, the ACI_n_ ranges from 0.2 to 0.22 in line with what was found in clean air Arctic summer conditions (Koike et al., 2019).

In summary, the microphysical properties of marine stratiform clouds advected over the NEA Ocean readily respond to changes in aerosol number concentration below the cloud base. This demonstrates that marine aerosols may significantly impact the microphysical properties of marine clouds over the NEA Ocean being an integral part of the oceanic biota‐cloud‐climate feedback system.

### 3.3 Aerosol Impact on Cloud Optical Properties Mediated by CDNC

It is well established that cloud albedo and COD in liquid clouds are primarily a function of liquid water path (Frey et al., 2017), therefore any modifications of cloud optical properties attributable to aerosol‐induced microphysical perturbations must be evaluated along with possible adjustments of cloud macrophysical properties. We have studied the relationship of cloud geometrical parameters (H_base_, H_top_ and H_thick_), LWC, albedo, and COD retrieved at MHD by the SYRSOC algorithm with N_a_ and CHL, along with the main meteorological parameters controlling the occurrence of stratiform clouds in this area of the world ocean (Table 1). The ECMWF‐ERA5 accessed from the Copernicus Climate Change Service was used to perform spatial correlation analysis, similar to that done with CHL. No time‐lag effect on the correlations between cloud parameters and meteorological variables was found. The resulting correlation maps are shown in Figures S12‐S16 in Supporting Information S1. The maps show that correlations, when present, are evident only in a region close to MHD (50°−55°N and 10°−15°W; dashed box in the presented maps, hereafter referred to by MHD box) and do not extend over the oceanic region identified by the relationship with CHL.

**Table 1:** Correlation Coefficients Between Clean Marine Cloud Data Measured at MHD and Meteorological Variables

Variable	T	SST	RH	PVV	LTS	CHL	N_a_
R_eff_	***−0.33***	***−0.28***				***−0.46***	***−0.50***
CDNC	*0.27*	0. 22			*0.27*	***0.44***	***0.65***
H_base_			***−0.72***		***0.75***	0.23	***0.41***
H_top_		0.20	***−0.71***		***0.74***	0.21	***0.38***
H_thick_			*0.25*	***−0.32***	*−0.24*		−0.23
Albedo			***0.50***		***−0.37***		
|Albedo|			***0.55***		***−0.30***		
COD			***0.44***		***−0.29***		
|COD|			***0.54***		***−0.29***		

*Note.* Correlations with CHL and N_a_ were added for comparison. Coefficients reported in bold are statistically significant at *p < 0.05*, while those in italics are statistically significant at *p < 0.1*. The sign | | indicates data normalized to cloud thickness. Coefficients associated with *p > 0.2* have not been reported.

It can be inferred from Table 1 that cloud microphysical properties (R_eff_ and CDNC) are mainly controlled by CHL (proxy of the oceanic biological activity) and aerosol number concentration, with a minor association to T and/or SST (which both tend to co‐vary with marine biological activity). Conversely, Cloud macrophysical (H_base_, H_top_, and H_thick_) and optical properties (COD and albedo) are mainly influenced by meteorological conditions such as RH and LTS, as expected, with no apparent linear dependence on either temperature or biological activity. Increased RH (more water available in the atmosphere) is associated with lower H_base_ and H_top_ values, due to rapid activation of cloud nuclei, as well as to enhanced cloud albedo and COD. The parameters most influenced by LTS are H_base_ and H_top_, which tend to increase in unstable conditions (deeper boundary layer), in agreement with the results from both observational (Wood & Bretherton, 2006) and modeling (Bretherton et al., 2013) studies.

CHL and N_a_ do not show significant linear correlations with the SYRSOC retrieved cloud albedo, nor with the normalized albedo (albedo/cloud thickness). This suggests that, although there is a noticeable effect on the cloud microphysical properties, the emissions from the marine biota are not linearly related to cloud reflectivity. On the other hand, the effect of cloud microphysics on the normalized optical properties (albedo and COD) is significant (*p* < 0.05; Figure S17 in Supporting Information S1). The correlation coefficients between CDNC and normalized albedo (normalized COD) are equal to 0.50 (0.43). Our data show that the most important microphysical parameter in controlling cloud albedo is LWC, in agreement with previous studies (Frey et al., 2017; Liu et al., 2020). LWC accounts for up to 53% (*R* = 0.73; slope = 0.85; intercept = 0.06) of the albedo variance (Figure S18 in Supporting Information S1). Past studies have shown that aerosol effects on cloud albedo are mediated through a chain of cloud microphysical and macrophysical properties, all linked by two‐way feedback. Recently a summary of potential (and sometimes contrasting) mechanisms relevant to marine stratocumulus clouds were provided by Glassmeier et al. (2021). Depending on relative humidity, the occurrence of precipitations, entrainment rate, and CDNC concentrations, the sensitivity of cloud albedo to aerosol perturbations can vary from positive to negative. As a result, for the same geographical area, both positive and negative sensitivities to CDNC can be found. Such sensitivities can be quantified, on a statistical basis, using the joint probability histograms approach (Gryspeerdt et al., 2019). We utilized the joint histograms data analysis approach on the SYRSOC dataset from MHD to investigate the dependence of LWC and albedo on CDNC. The use of joint histograms is preferable in this case, with respect to a simple linear regression, as it can evidence also nonlinear relationships between CDNC and LWC or albedo and account for dispersion of the data caused by the variability in the boundary (meteorological) conditions, hence providing more accurate information about the shape of the relationship. This approach has been previously used to analyze the dependence of cloud fraction (Gryspeerdt et al., 2016) and albedo (Gryspeerdt et al., 2017) on aerosol properties.

**Figure 3:**
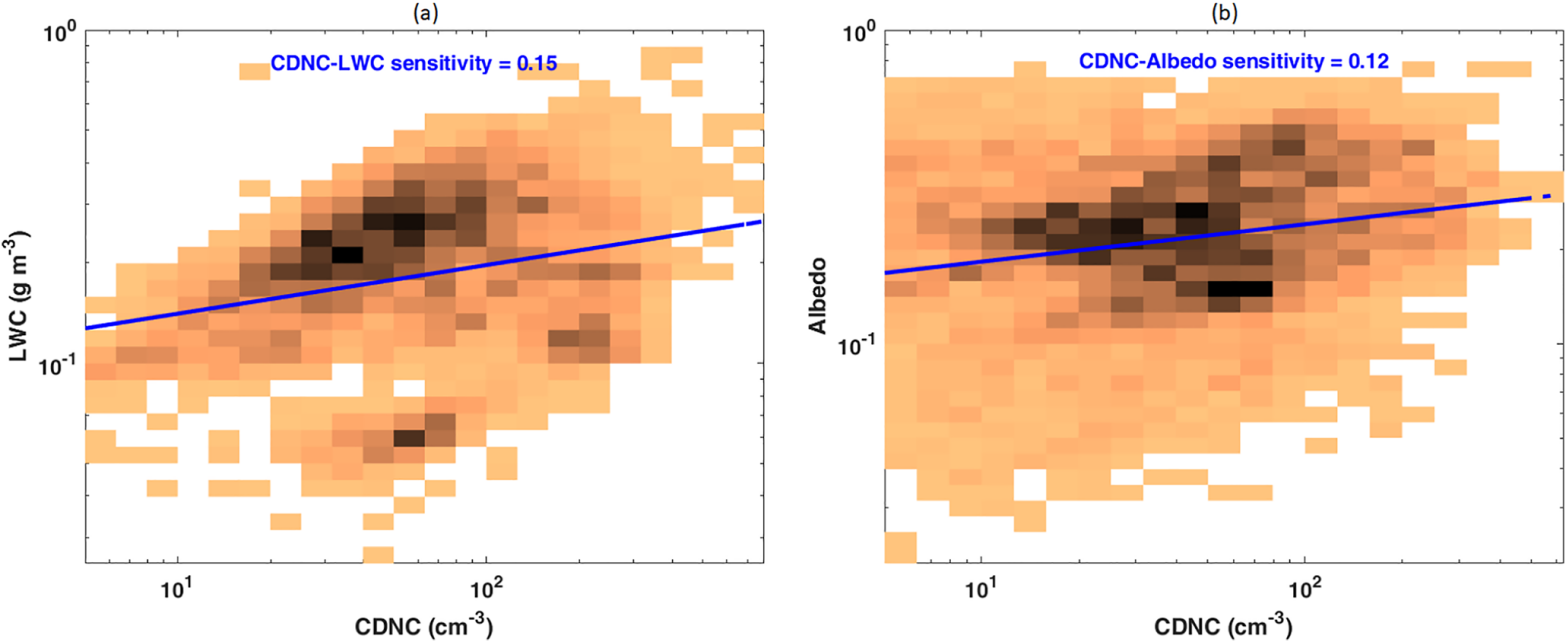
Joint probability histograms of (a) cloud droplet number concentration – liquid water content (CDNC‐LWC) and (b) CDNC‐Albedo; darker colors indicate higher probability; the colors are normalized so that the sum of the pixels is one in each plot. The blue line is the linear regression fit on the log‐log data and the linear sensitivity is inserted at the top of each plot. Sensitivities were calculated as the slope of the linear regression coefficient in the log‐log scale.

The CDNC‐LWC and CDNC‐albedo joint histograms (Figure 3) and the log‐log linear fit lines show that such relationships are mostly non‐linear and positive. An increase in the LWC and albedo with increasing CDNC is observed particularly in the bin range of 20–200 cm^−3^ (low CDNC) and in the range of LWC from 0.1 to 0.5 g m^−3^, suggesting that increases of marine biogenic aerosol concentrations lead, on average, to LWC and albedo enhancements in stratiform clouds over the NEA Ocean. Positive adjustments of cloud LWC to aerosol perturbations are commonly explained by increased colloidal stability of clouds of reduced droplet size with a consequent effect of precipitation suppression (see, the discussion below). It should be noted, however, that joint histograms only provide a statistical representation of the relationship between cloud properties and aerosols and only allow us to infer the possible mechanisms involved in such a relationship. This is the first application of joint histograms to the analysis of susceptibility of cloud properties to aerosols on the basis of ground‐based cloud retrievals. With aim of comparison with analogous representations based on global satellite observations, the analysis of Gryspeerdt et al. (2019) also shows positive adjustments of liquid water path to CDNC perturbations in the low concentrations range (10–30 cm^−3^) turning positive at higher CDNC. Our study suggests that in the NEA Ocean, positive adjustments can be associated with CDNC changes extending beyond 100 cm^−3^, hence to a wider range compared to previous reports.

## 4 Discussion

By using a unique dataset of multi‐year (2009‐2015) remote sensing cloud observations, the effect of the oceanic biological activity on cloud properties was investigated over the NEA Ocean. The retrieved cloud microphysical parameters fall in the same range as those obtained from satellite (MODIS) observations (Figure S2 in Supporting Information S1) – widely used in previous studies – but do not require assumptions of adiabaticity and are much less affected by common retrieval errors affecting satellite cloud observations (e.g., from the solar zenith angle; Gryspeerdt et al. [2019]). This allows us to investigate the subtle ACI effects on cloud properties at the low aerosol concentration domain typical of marine background conditions.

**Figure 4:**
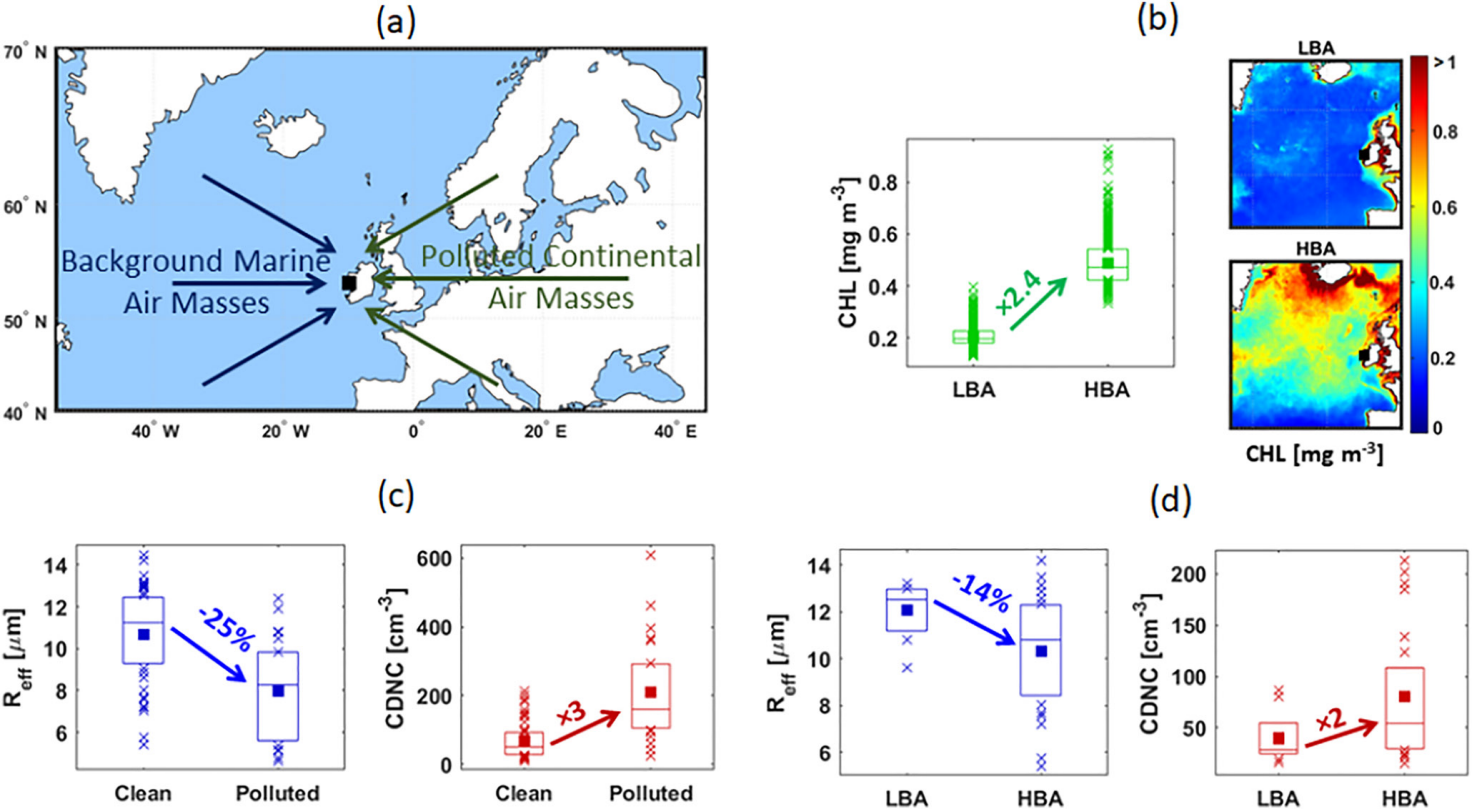
(a) The direction of background marine and polluted continental air masses arriving at Mace Head station. (b) Seasonal average sea surface chlorophyll a concentration during 2009‐2015, illustrating low biological activity in the Northeast Atlantic waters during winter (LBA) and high activity in summer (HBA). (c) Quantification of change in cloud microphysical properties passing from Polluted to Clean marine air masses. (d) Quantification of change in cloud microphysical properties passing from LBA to HBA.

Our data show that the oceanic biota affects marine cloud microphysical properties by affecting the aerosol number concentration in the MBL. The effect is a reduction of R_eff_ coupled to enhanced CDNC during periods of HBA, compared to the winter baseline period. The ACI index shows that marine stratiform clouds, over the NEA, are sensitive to aerosol number concentration variations supporting the above findings. These results are consistent with the recent findings by Rosenfeld et al. (2019), which concluded that the variability in aerosol concentration can explain ∼45% of the variability of the cloud radiative effect of oceanic low‐level clouds over the Southern Ocean, using satellite observations. Within the range of variability encountered in our dataset, ranging from LBA to HBA conditions (winter to summer) induces an increase in surface CHL of ∼2.4 times in seawaters facing MHD, associated with a doubling in CDNC and a decrease of R_eff_ by ∼14% (Figure 4).

Quantifying the aerosol effects on cloud albedo is less straightforward due to the complexity of the relationship between cloud reflectivity and microphysical properties. The LWC sensitivity to CDNC is important since it may enhance or offset the overall albedo sensitivity (Glassmeier et al., 2021). The observed positive adjustments of stratiform cloud macrophysics to aerosol perturbations can be partly explained by precipitation suppression effects (Albrecht, 1989). Smaller, more numerous droplets can diminish collision coalescence and delay precipitation onset (Christensen et al., 2020); longer‐lived clouds have a stronger cooling effect on climate. Precipitation is considered as strongly suppressed when R_eff_ does not exceed 12–14 μm in marine low clouds (Fan et al., 2020; Freud & Rosenfeld, 2012). In our study, the R_eff_ was below 12 μm in 54% of the time steps (4,259 of 7,884 data points) during LBA and in 72% of the time steps (24,230 of 33,808 data points) during the HBA. The actual role played by aerosol‐induced precipitation suppression associated with marine biogenic emissions in the NEA deserves further studies.

The recent literature focusing on cloud adjustments to marine aerosol perturbations provides a wide range (from negative to positive) sensitivity. A positive LWC sensitivity is more common at small CDNC values, while the sign turns negative above a certain threshold which depends on the cloud system and on meteorological constraints (Glassmeier et al., 2021). As expected, and in agreement with the results of satellite observations of marine clouds in the Southern Ocean (Rosenfeld et al., 2019) and the Northwest Atlantic Ocean (Sinclair et al., 2020), our findings show that the variability of cloud macrophysical properties is primarily dictated by meteorological conditions (Table 1). Nevertheless, our analysis demonstrates a positive non‐linear relationship between CDNC and both cloud LWC and albedo (Figure 3). In consistency with our findings, Michibata et al. (2016) showed a positive sensitivity of liquid water path to CDNC over the NEA Ocean employing satellite data (A‐Train). However, satellite observations (Chen et al., 2014; Gryspeerdt et al., 2019) and global model simulations (Lebo & Feingold, 2014; Sato et al., 2018) reported a negative response of liquid water path to CDNC, as a result of enhanced evaporation‐entrainment feedbacks (Ackerman et al., 2004; Wang et al., 2003; Xue et al., 2008), which unreels to some extent the cloud albedo effect. In spite of such a large span in marine cloud susceptibilities to aerosol perturbations, most studies relying on global satellite observations consistently report more negative adjustments in the tropics with respect to the mid‐latitudes (Gryspeerdt et al., 2019; Michibata et al., 2016). The actual range of aerosol concentrations relevant for actual positive cloud adjustments is a matter of debate. Our study suggests that perturbations in CDNC over a wide range, from 20 to 200 cm^−3^ propagate to positive adjustments in stratus cloud LWC and albedo in the NEA, hence supporting the importance of mechanisms associated with precipitation suppression.

Our study supports the importance of aerosol‐cloud interactions in the marine atmosphere triggered by the emissions from the marine biota. Variations in cloud microphysical properties associated with marine aerosol emissions and similar to the ones observed in this study have been associated with significant radiative effects in the literature. Observations in the Southern Ocean, typically considered a unique clean marine environment for its remoteness, showed that ocean biology can double the CDNC over biologically productive waters in summer, with respect to unproductive waters (McCoy et al., 2015). It was calculated that such a change in CDNC can cause a net reflection in shortwave radiation between −4 and −6 W m^−2^ as an annual average, exceeding −10 W m^−2^ in summer. A similar doubling in CDNC has been reported (Meskhidze & Nenes, 2006) over a blooming area of the Southern Ocean with respect to non‐blooming regions, associated with a more intense reduction of R_eff_ (∼30%); such variation in cloud microphysical properties was estimated to cause a strong cooling reaching −15 W m^−2^. The aforementioned values are roughly equivalent to the yearly mean radiative forcing estimated in global circulation models as a result of aerosol‐cloud interaction over severely polluted regions (Bian & Prather, 2002; Jones et al., 1994; Menon et al., 2002; Rap et al., 2013; Zelinka et al., 2014). Further studies in the Southern Ocean quantified the effect of secondary sulfate emissions on the annual mean reflected shortwave radiation to be −9.32 W m^−2^ (Thomas et al., 2010), as a result of a 15%–18% reduction in R_eff_. To this same seasonal cycle of R_eff_ over the Southern Ocean, McCoy et al. (2014) attributed a difference in the reflection of the shortwave radiation of up to −8 W m^−2^.

As observed from the MHD location, the NEA can be considered pristine 64% of the time (Grigas et al., 2017). In other model simulation studies, the occurrence of pristine air masses over the NEA is estimated to be even lower (Hamilton et al., 2014). Consequently, one can argue that anthropogenic aerosol particles may have a much stronger impact on cloud microphysical properties than biogenic ones. By comparing background marine conditions to anthropogenically influenced cases (Figure 4 and Table S2 in Supporting Information S1), we observed an enhancement of CDNC by 3 times and a 25% reduction in R_eff_, passing from unperturbed marine clouds to polluted conditions. These variations are not far from those observed passing from background LBA to HBA conditions, doubling in CDNC and decreasing in R_eff_ by ∼14% (Figure 4). These findings suggest that, over the NEA ocean, the impact of oceanic biological activity on cloud properties may be of similar magnitude to that exerted by anthropogenic and continental aerosols and, in any case, not negligible in comparison.

## 5 Conclusions

This work investigates the impact of oceanic biological activity on the properties of stratiform clouds over the NEA using cloud remote sensing observations at Mace Head from 2009 to 2015. Our analysis indicates the potential of accurate remote‐sensed cloud parametefrom ground‐based measurements for aerosol‐cloud radiative effect studies. The main finding of the present work is that marine biota affects cloud microphysical properties by diminishing (−14%) cloud droplet effective radius (R_eff_) and enhancing (+100%) CDNC during periods of enhanced biological activity with respect to the quiescent wintertime. This occurs because the oceanic biological activity influences the concentration of aerosol particles, in the CCN relevant‐size range, in the unperturbed marine boundary layer.

Cloud macrophysical properties are mainly controlled by meteorological constraints (tropospheric stability and water vapor availability), and the variability of cloud albedo is primarily dictated by changes in cloud liquid water content. Anyhow, after normalizing for the meteorological constraints, aerosol perturbations on cloud liquid water content and cloud albedo are detectable, and such response results are positive over a wide range of CDNC, and higher with respect to the outcomes of previous studies based on global analyses. It follows that the phytoplankton‐induced enhancement in the CCN concentration, occurring during spring‐summer over the NEA Ocean, contributes to generally brighter stratiform clouds.

Variations in cloud microphysical properties similar to the ones observed in this study have been associated with significant radiative effects over the Southern Ocean. This suggests that the net radiative effect of the observed winter‐summer variation in cloud properties may not be negligible over the NEA either. Along the same line, the magnitude of the variation in cloud microphysical properties between high and low oceanic biological activity periods is comparable to that between anthropogenically influenced and pristine marine air masses. This implies that, over the NEA, the seasonal CCN fluctuations due to biological activity may have a similar radiative impact as those caused by the advection of anthropogenic continental aerosols.

The uncertainty of climate models associated with aerosol‐cloud interactions is largely due to the lack of accurate parameterizations and the limited understanding of the complex feedbacks. Our results demonstrate an evident impact of the oceanic biota on marine stratiform cloud properties over the NEA, where radiative forcing is traditionally considered anthropogenically driven in climate models. Our study indicates that CCN variability impacted by marine biogenic emissions can be a key factor to constrain to reducing the uncertainty of radiative forcing from aerosol‐cloud interactions over the global ocean.

## Conflict of Interest

The authors declare no conflicts of interest relevant to this study.

## Supporting information

Supporting Information S1

## Data Availability

The SYRSOC results for the 118 cloud cases can be accessed at http://doi.org/10.5281/zenodo.154137. Measurement times of the selected 52 background marine cases are shown in Data S1 (supplementary materials). The remote‐sensing CHL dataset is available at http://marine.copernicus.eu/ (Identifier: OCEANCOLOUR_GLO_CHL_L4_REP_OBSERVATIONS_009_082). The ERA5 meteorological dataset can be accessed at https://cds.climate.copernicus.eu/. The air mass back‐trajectories data are archived through https://www.arl.noaa.gov/. The codes used in this study for data elaborations are available from GitHub at https://github.com/karam-mansour/Phytoplankton_Cloud_North_Atlantic and can be cited through https://doi.org/10.5281/zenodo.6489668.
