# Supplementary material for: Phytoplankton Impact on Marine Cloud Microphysical Properties Over the Northeast Atlantic Ocean
Source: J Geophys Res Atmos. 2022 May 24;127(10):e2021JD036355. doi: 10.1029/2021JD036355 (PMC9285769; doi:10.1029/2021JD036355)
Supplement: Supplementary file 1 — Supporting Information S1 [file JGRD-127-0-s001.pdf]

AGU PUBLICATIONS

Journal of Geophysical Research: Atmospheres

Supporting Information for

Phytoplankton Impact on Marine Cloud Microphysical Properties over the Northeast Atlantic Ocean

Karam Mansour[1,2], Matteo Rinaldi[1], Jana Preißler[3], Stefano Decesari[1], Jurgita Ovadnevaite[4], Darius Ceburnis[4], Marco Paglione[1], Maria C. Facchini[1], and Colin O'Dowd[4]

[1]Italian National Research Council - Institute of Atmospheric Sciences and Climate (CNR-ISAC), Bologna 40129, Italy.

[2]Oceanography Department, Faculty of Science, Alexandria University, Alexandria 21500, Egypt.

[3]Leosphere, 91401 Saclay, France.

[4]School of Physics, Ryan Institute's Centre for Climate and Air Pollution Studies, National University of Ireland Galway, Galway, Ireland.

Contents of this file

Text S1 to S2
Figures S1 to S18
Tables S1 to S2
Data Set S1

Introduction

This file contains 2 supplementary texts explaining the detailed method to compare SYRSOC and MODIS cloud measurements at Mace Head as well as the detailed method of concentration weighted trajectory model. In addition, 16 graphs and 2 tables support the main text.

Text S1. Comparison of SYRSOC and MODIS cloud data at MHD

The Moderate-Resolution Imaging Spectroradiometer (MODIS) level-2 cloud product from both Terra (MOD06) and Aqua (MYD06) platforms (Platnick et al., 2017) were used to compare with SYRSOC output. MODIS has several spectral channels of which near-infrared band, including window channels centred near 1.6 and 2.1 μm, in addition to an AVHRR heritage 3.7 μm channel. These provide cloud microphysical information at 1-km horizontal resolution retrievals and temporal period of collection (every 5 minutes along the orbit track). One 5 min file, referred to as data granule, contains data from roughly 1354 1-km pixels across-track to 2030 km along-track of Earth located data. Thus, a data granule is comprised of approximately 2.7 M 1-km pixels.

We analyzed the granules from both Terra and Aqua which were coincident with the SYRSOC clean marine cases at MHD. This study is based on the 3.7 μm channel, which is less affected by retrieval biases than others (Grosvenor et al., 2018). Out of the 52 clean marine datasets distributed over 47 days (47×2×288 = 27,072 granules), 443 granules have an overpass within 6 hours around the mid-time of SYRSOC measurements. We selected a time difference threshold of 6 hours to maintain conditions similar to SYRSOC measurement, without losing too many data points. In each granule, the two external edge scenes were excluded for data quality (Rosenfeld et al., 2019). To minimize the uncertainty, pixels within each granule that have the following conditions are considered:

i. Liquid water–phase clouds.
ii. One cloud layer.
iii. Solar zenith angle (SZA) less than 65°, due to the reliability of retrieved cloud properties starts deteriorating with increasing SZA (Grosvenor & Wood, 2014).
iv. Highest 50% of cloud optical depth (COD) within the granule.

MODIS could not retrieve CDNC directly. To obtain CDNC, the input obtained from MODIS cloud products of R_{eff} and COD was utilized to solve the following equation under the abovementioned conditions.

$$CDNC = \frac{10^{1/2}\, {C_w}^{1/2}\, COD^{1/2}}{4\pi\, {\rho_w}^{1/2}} (\frac{R_{eff}}{k})^{-5/2}$$

where k is a constant factor that relates R_{eff} to the mean volume radius and equal to 1.08 (Freud & Rosenfeld, 2012). ρ_w is the liquid water density. C_w is the rate of increase of LWC with height for a moist adiabatic ascent and is referred to as the condensation rate (Brenguier et al., 2000) or the water content lapse rate (Painemal & Zuidema, 2011). C_w is a weak function of temperature and pressure, varying between 1.8 and 2.25 $\times 10^{-3}$ g m^{-3} m^{-1} in the temperature range 280–290 K at 980 hPa (Szczodrak, Austin, & Krummel, 2001). In our study, we assumed C_w is a constant equal to 2×10^{-3} gm m^{-3} m^{-1}. Grosvenor & Wood (2014) estimates a 2% of CDNC underestimation if C_w assumed to be constant throughout the cloud instead of taking into account the temperature and pressure variation and smaller errors for shallower clouds.

MODIS data were averaged over an area from 52.33 to 54.33 °N and from 9.90 to 11.90 °W (2° lat/long from MHD toward the Ocean). Out of the 52 clean marine datasets, 27 cases were coincident with overpasses of the Aqua or Terra satellite over MHD and within 6 hours before and after the mid-time of SYRSOC cases.

The comparisons are shown in Figure S2. The R_{eff} by SYRSOC is slightly lower than that by MODIS, consistently with Preissler et al. (2016). CDNC is more scattered, consistently with the fact that it is still a very uncertain parameter (Grosvenor et al., 2018), nevertheless, the data fall around the 1:1 line.

Text S2. Three-dimensional Concentration Weighted Trajectory (3D-CWT)

The Concentration Weighted Trajectory (CWT) model is a tool for evaluating possible impacts of the long-range aerosol transport, by combining the residence time (trajectory points) of air masses over geographic regions with particle concentrations at a specific point (receptor site). Here, the three-dimensional CWT ($3D - CWT_{ijk}$) model was used to identify the NEA sea region (1) acting as an emission source of marine aerosols and (2) characterized by high CDNC and low R_{eff}. For each cloud case, five days (hourly time step) backward air mass trajectories reaching MHD at 100 m above ground level were computed, using the NOAA HYSPLIT model. HYSPLIT provides hourly trajectory points as a function of longitude, latitude, and altitude, representing the pathway of an incoming air parcel.

Corresponding to the studied 52 cloud cases, 6292 trajectory endpoints (52×121 hourly points per trajectory [120 h backwards plus one arrival point]) were distributed in the grid cells of a 1°×2° latitude/longitude resolution (latitude 33° to 80° N, longitude: 64° W to 08° E) which cover the NEA domain. A total of 1692 cells were obtained, of which 663 are non-empty cells in the limits of the area covered by back-trajectory endpoints. For a grid cell ij of the NEA domain, the weighted average concentration can be determined as:

$$3D - CWT_{ijk} = \frac{\sum_{t=1}^{L} C_t \, D_{ijkt}}{\sum_{t=1}^{L} D_{ijkt}}$$

Where t is the index of the trajectory corresponding to each cloud case, L is the total number of trajectories which is equal to 52 and C_t is the R_{eff} or CDNC value for each cloud case observed at the sampling location (MHD) on the arrival of trajectory t. D_{ijkt} is the number of trajectory points (air mass residence time) in the grid cell ij computed by excluding those exceeding the height value k, which is the observed cloud base height for the specific cloud case. Similarly, the previously mentioned equation was applied to in situ N_a (36 data points because N_a is not available for all cloud cases).

In general, the back trajectory arrival points (corresponding to MHD position) and the closest 3 points for each trajectory (3h before arrival time) were excluded from the analysis since they bias the statistics of both D_{ijkt} and CWT_{ijk}. Since the grid cells containing a low number of endpoints, extremely long distant trajectory points from MHD, can affect the robustness of CWT values, we removed each cell having D_{ijk} < median of D_{ijk} (less than 5 endpoints) among the non-empty ij cells. This reduces the number of considered cells to 378.

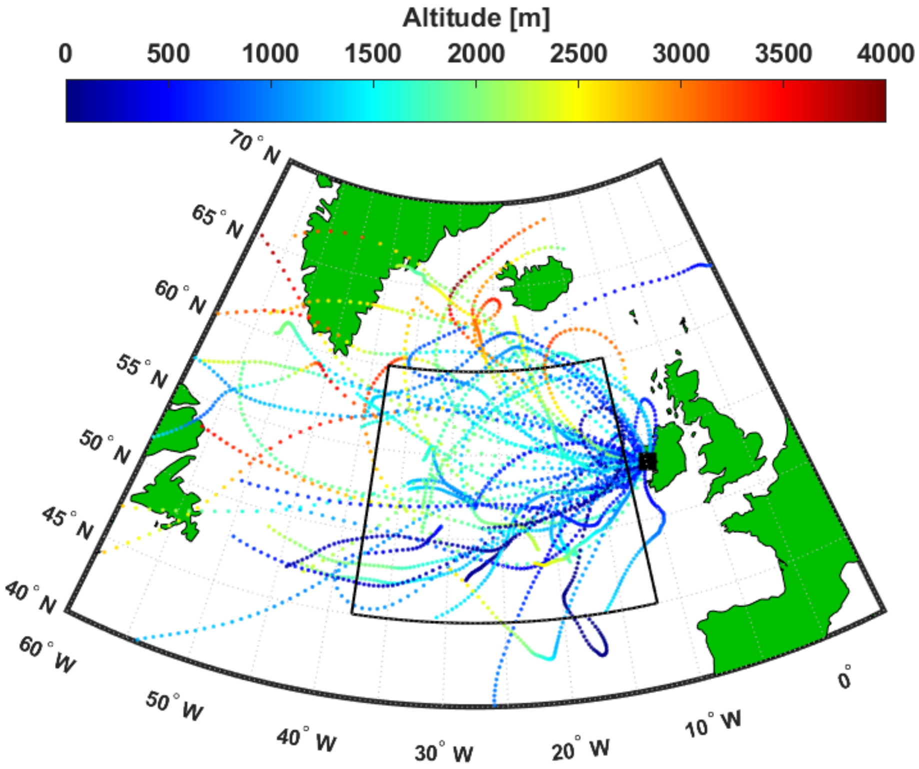

Figure S1. The 3-day air mass back-trajectories arriving MHD (represented by a filled black square) simultaneously to each cloud case at the cloud base altitude above the ground level during the clean marine conditions. The color scale represents the altitude of each endpoint of back-trajectories. The black box is the main area of interest of the present study.

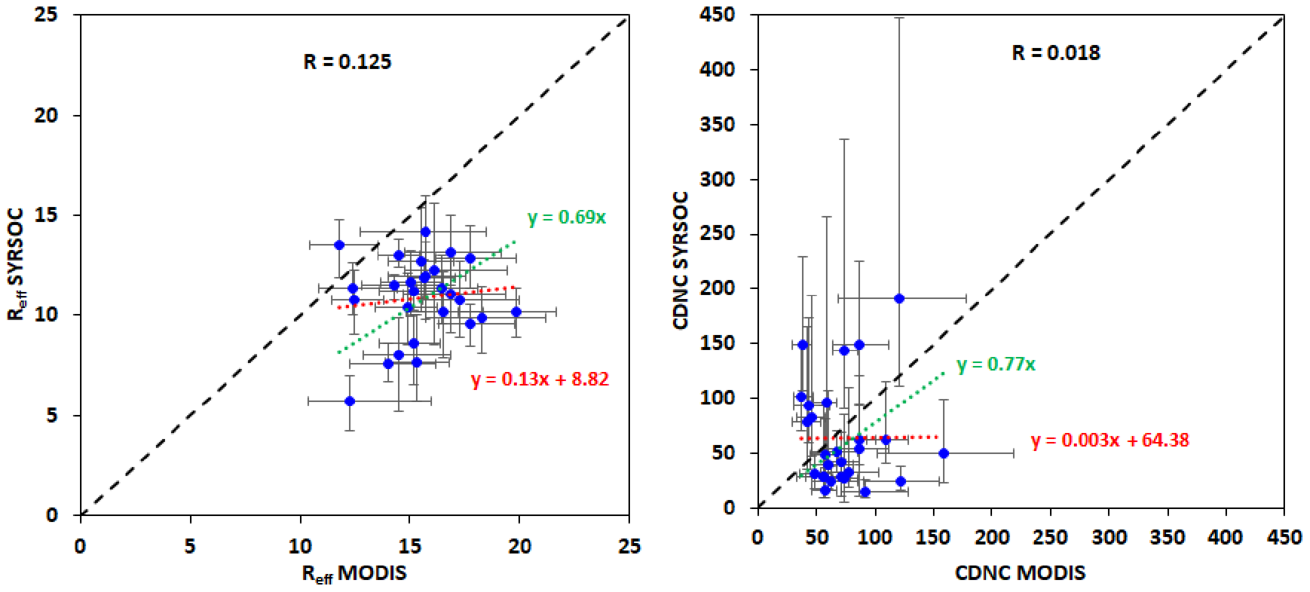

Figure S2. Comparison between MODIS and SYRSOC cloud microphysical properties (R_{eff} and CDNC). The red line is the linear regression fit whereas the green if the intercept is forced to zero. The vertical and horizontal bars extend to the 1st and 3rd quartiles of each cloud case. The 1:1 line (dashed black) is shown for reference.

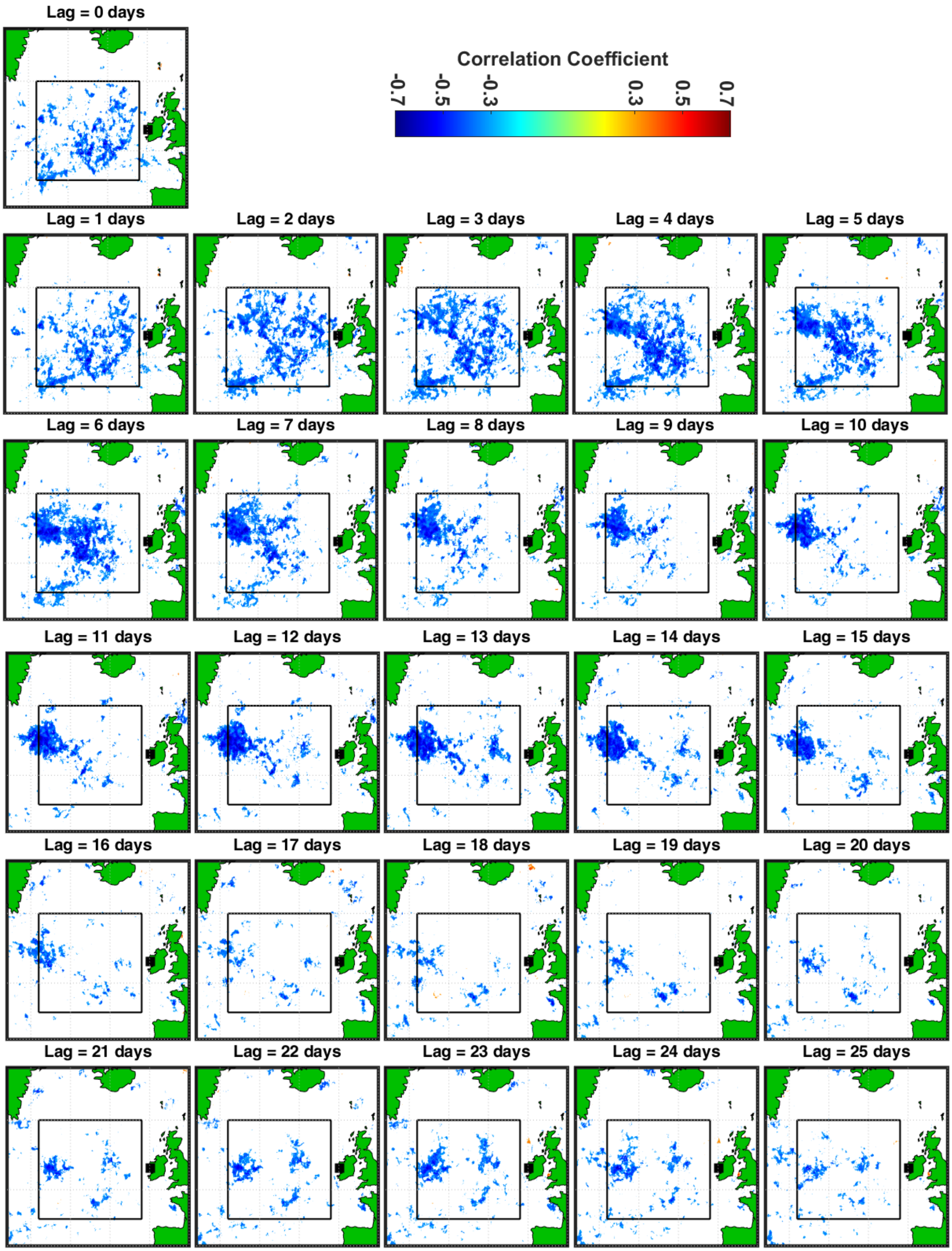

Figure S3. Spatial distributions of correlation coefficients between R_{eff} measured at MHD and CHL over the NEA Ocean at different time-lags from 0 to 25 days. Only significant correlation coefficients at a 95% confidence level are presented.

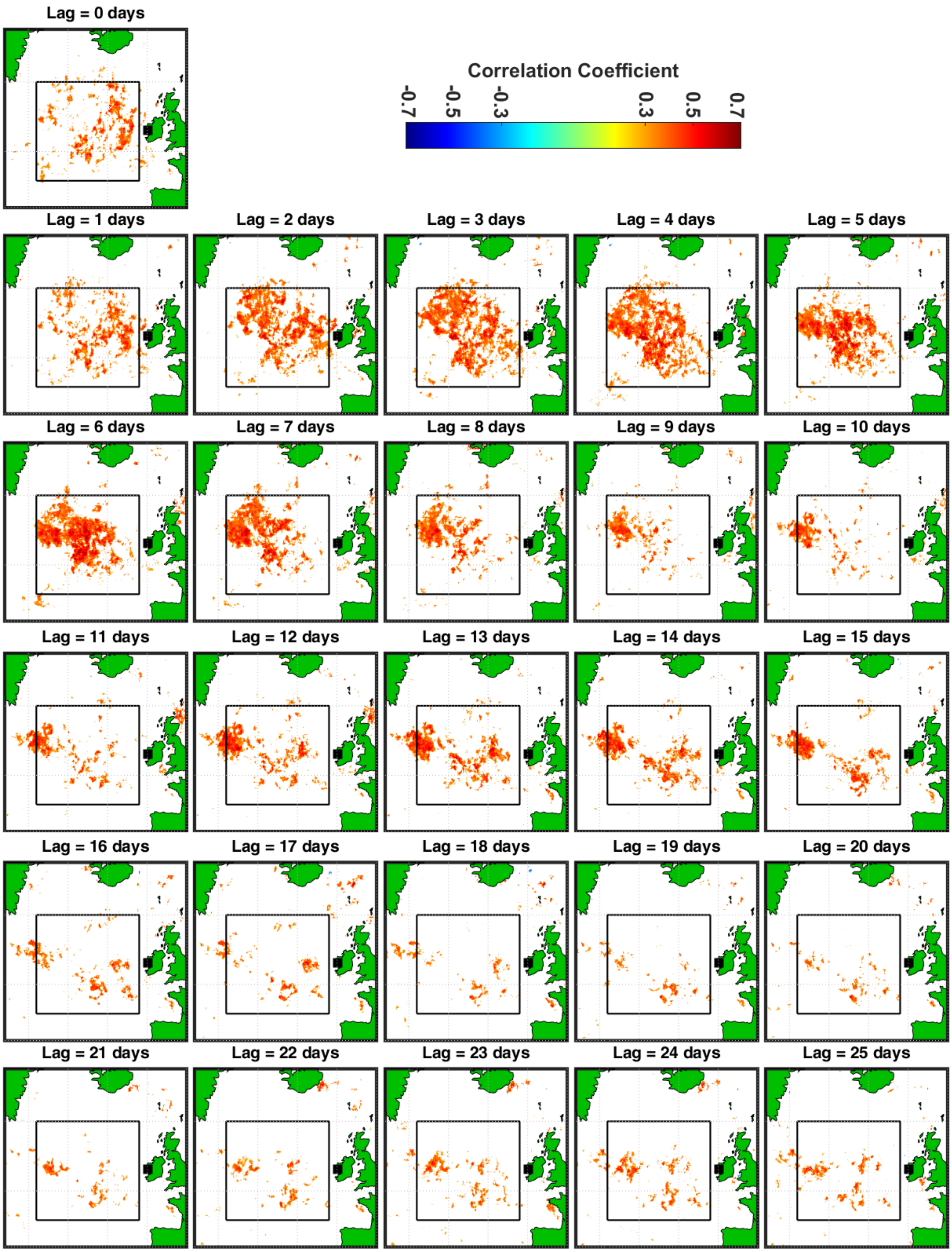

Figure S4. Same as Figure S3 but for CDNC.

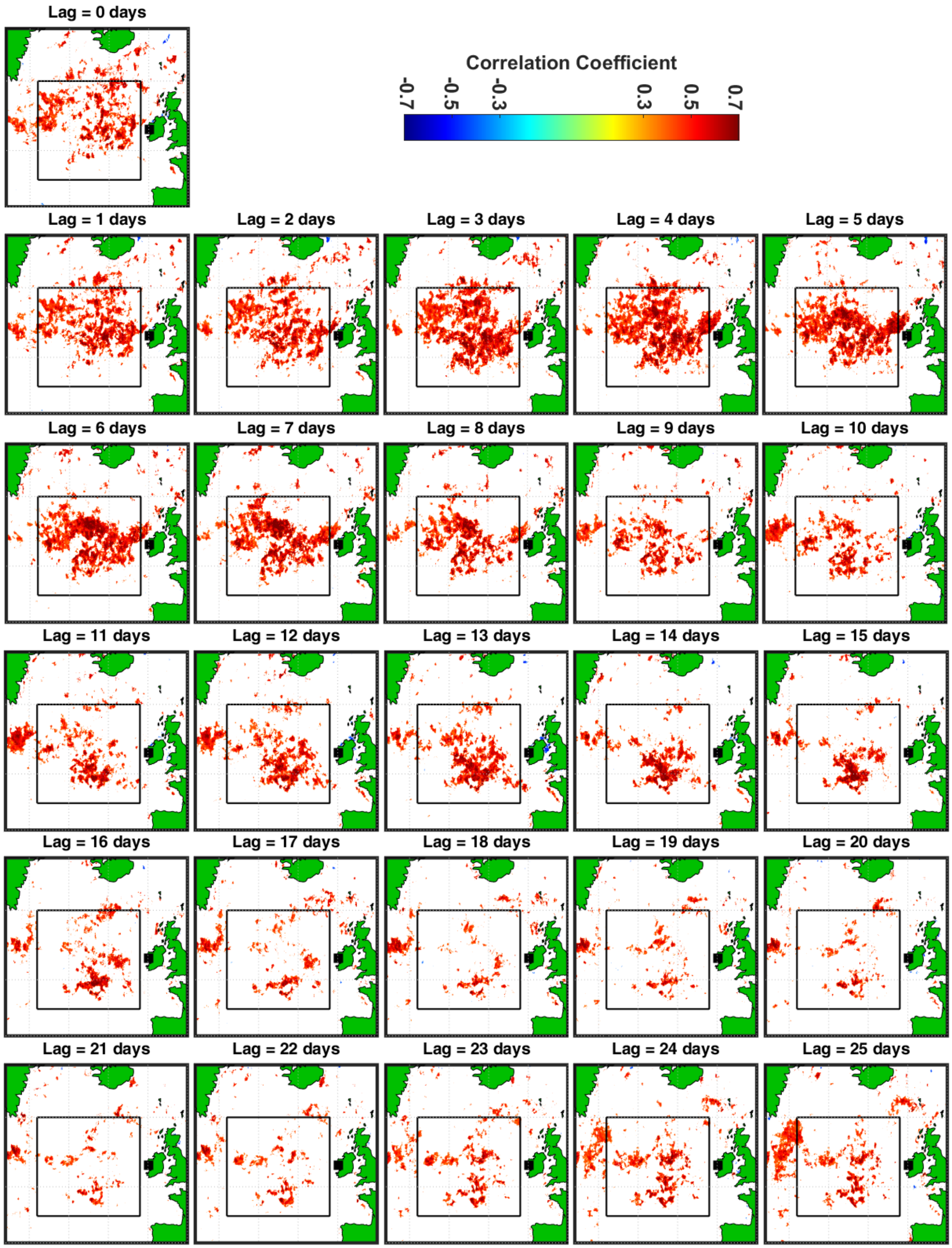

Figure S5. Same as Figure S3 but for N_a.

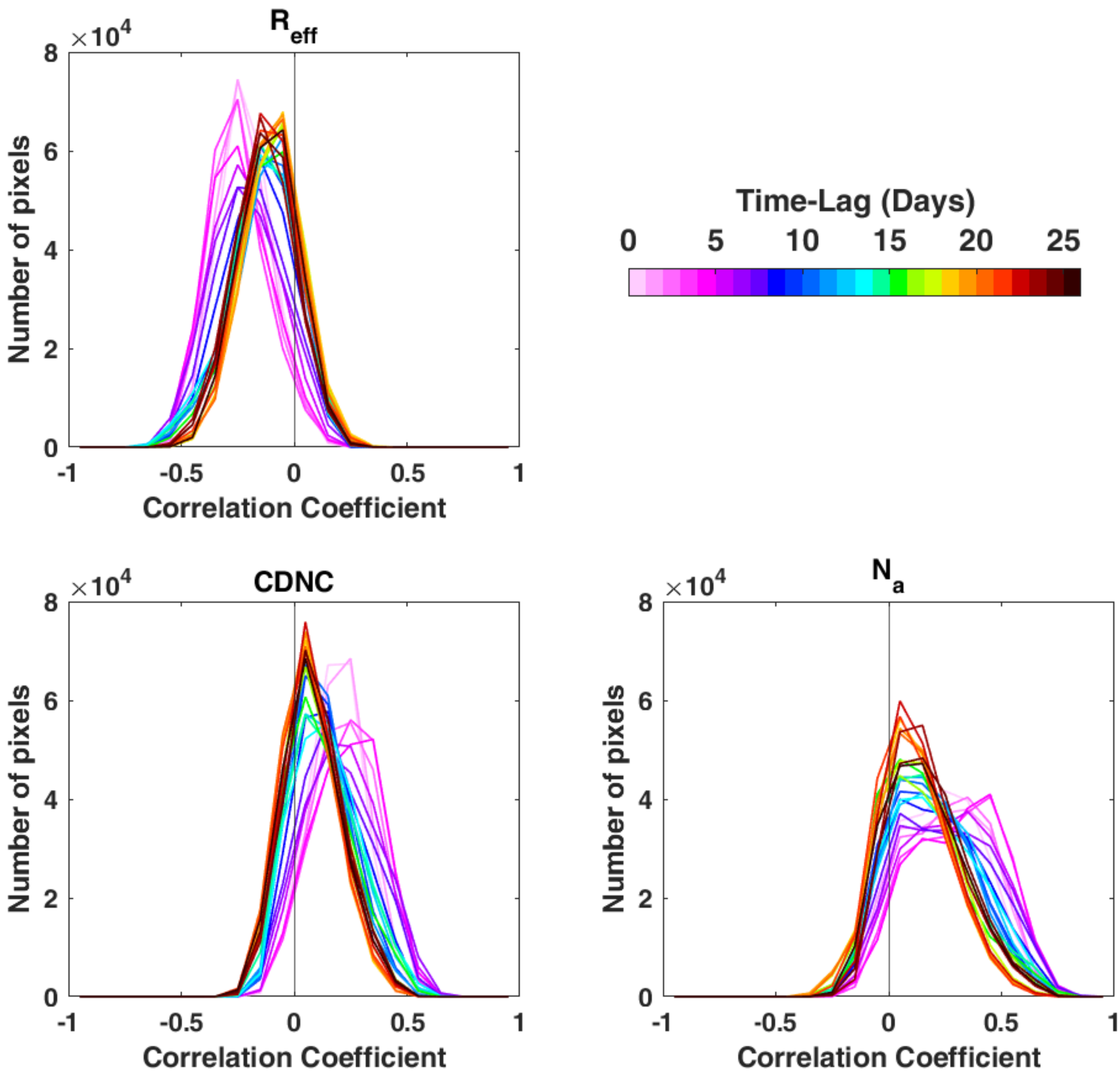

Figure S6. Correlation coefficient frequency distributions in the identified box (Figure 1) at different time-lags from 0 to 25 days.

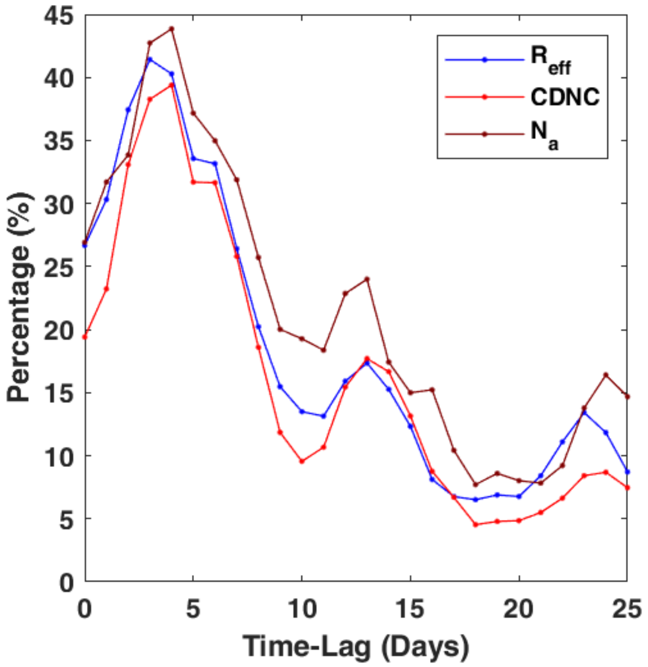

Figure S7. The percentages of the number of pixels with negative (positive) significant correlation for R_{eff} (CDNC and N_a) in the identified box (Figure 1).

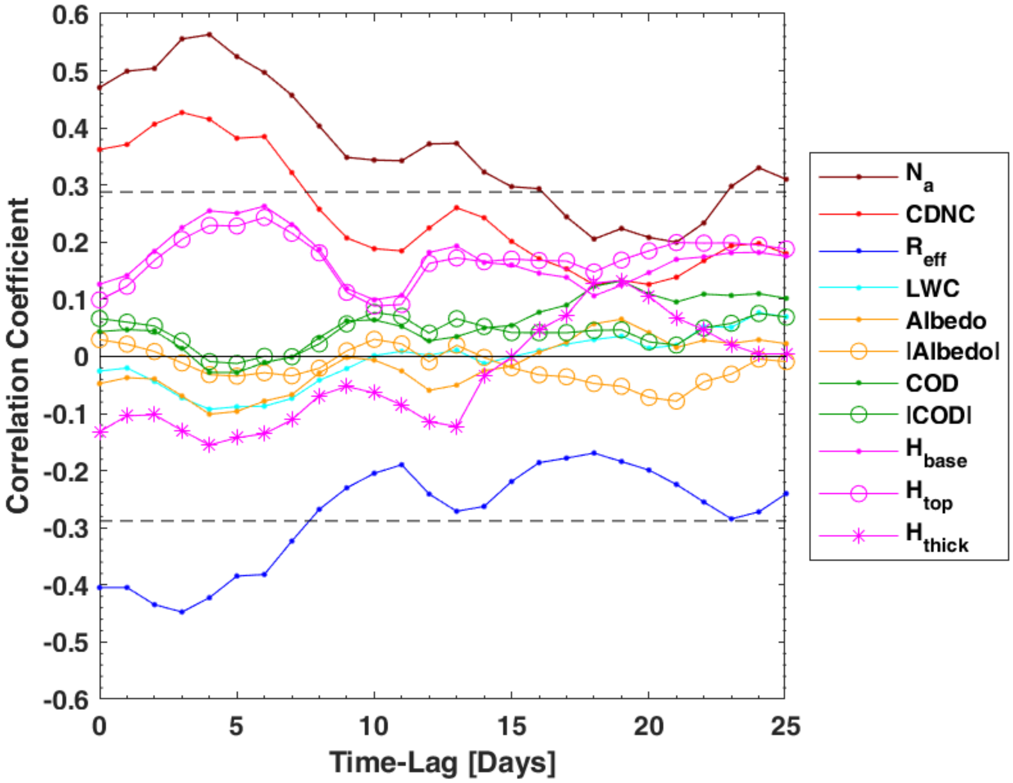

Figure S8. Correlation coefficients as a function of the time-lag between mean CHL within the identified box region and aerosol/ cloud properties. The black dashed horizontal lines represent the critical level of significant correlation ($p<0.05$). The sign || stands for normalized data. The correlations presented in this Figure are only indicative of the general correlation trends over the whole box region which includes correlating and non-correlating pixels due to the spatial variability of CHL.

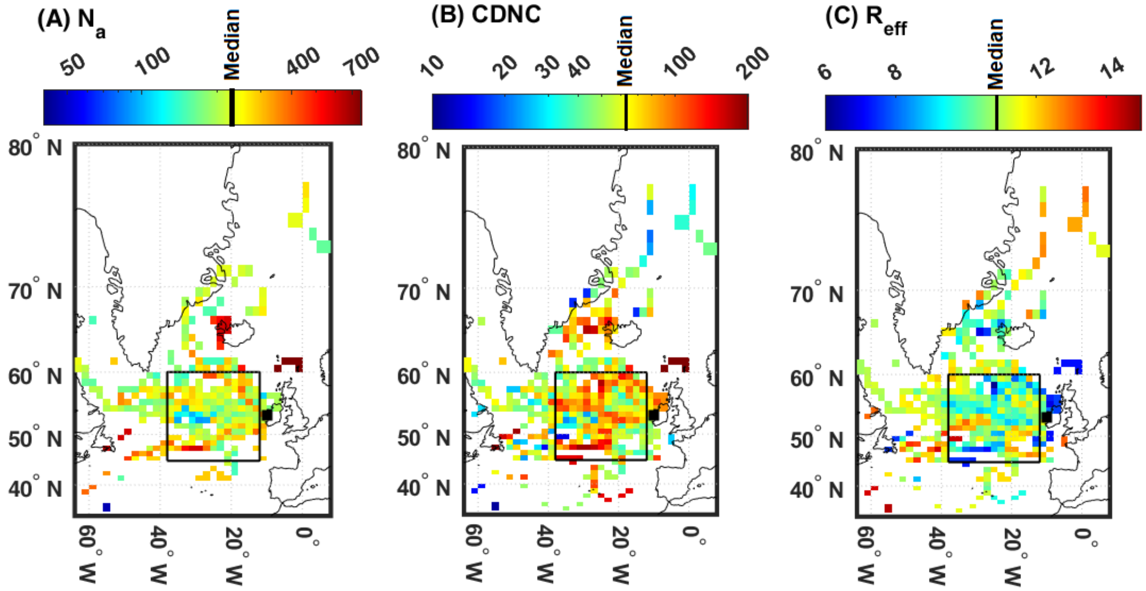

Figure S9. The 3D-CWT source maps for (A) N_a (B) CDNC and (C) R_{eff} dataset.

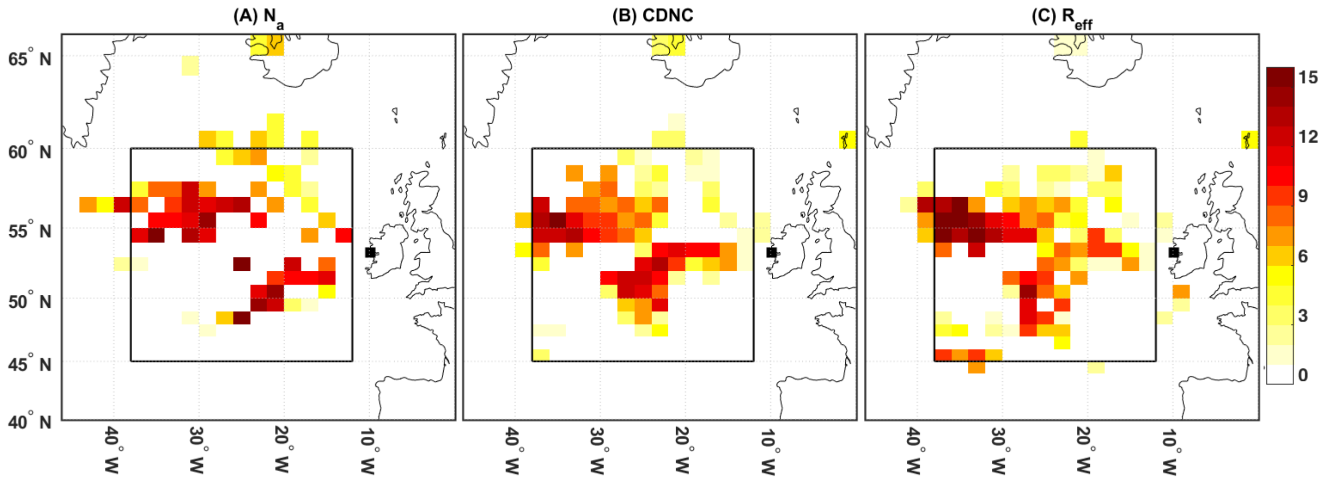

Figure S10. Oceanic regions have the highest probability of being a source of marine biogenic aerosol impacting cloud properties over the NEA. The colored pixels were identified by merging the results of the Spatio-temporal correlation analysis and the 3D-CWT model. The color scale reflects how many times a given pixel has CWT ≥ median (CWT ≤ median) and positive (negative) significant correlation coefficient in case of N_a and CDNC (R_{eff}) respectively, by running time-lag from 0 to 15 days.

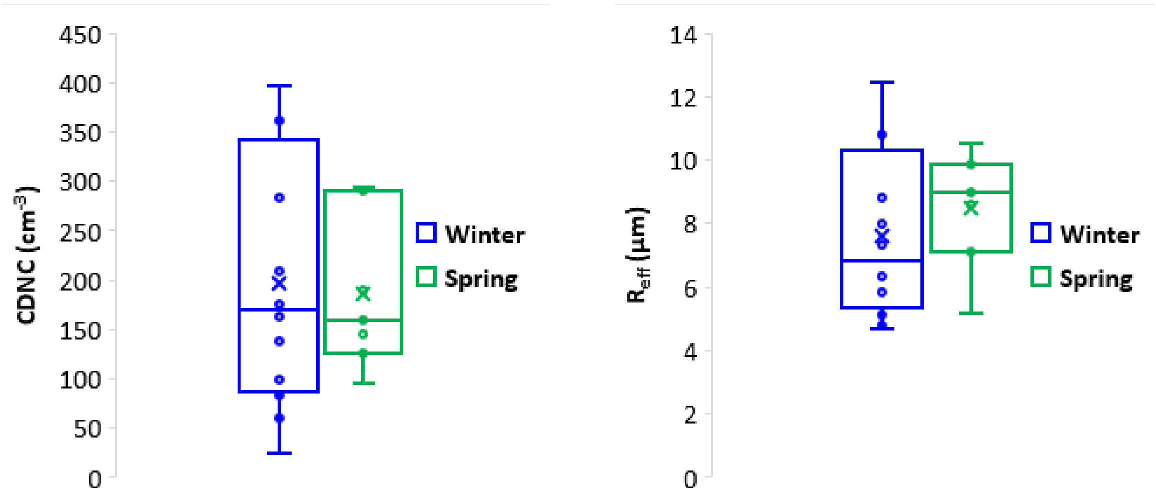

Figure S11. Quantification of seasonal variations in cloud microphysical properties of polluted cases. Winter represents a season of LBA (low biological activity) while spring represents a season of HBA (high biological activity) since the continental cases are absent in summer.

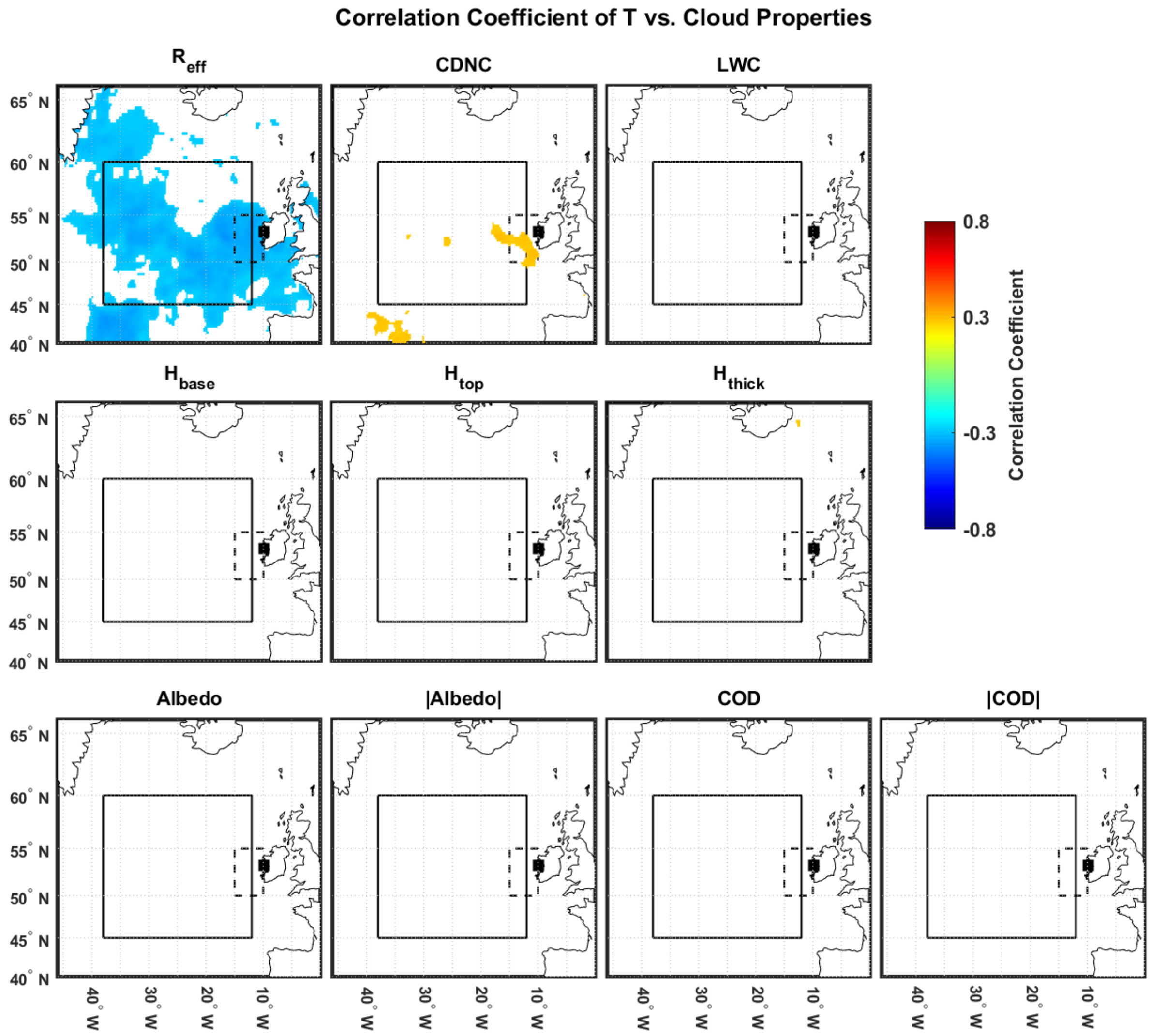

Figure S12. Spatial distributions of correlation coefficients between air temperature over the NEA Ocean and cloud properties measured at MHD. Only significant correlation coefficients (p<0.05) are presented.

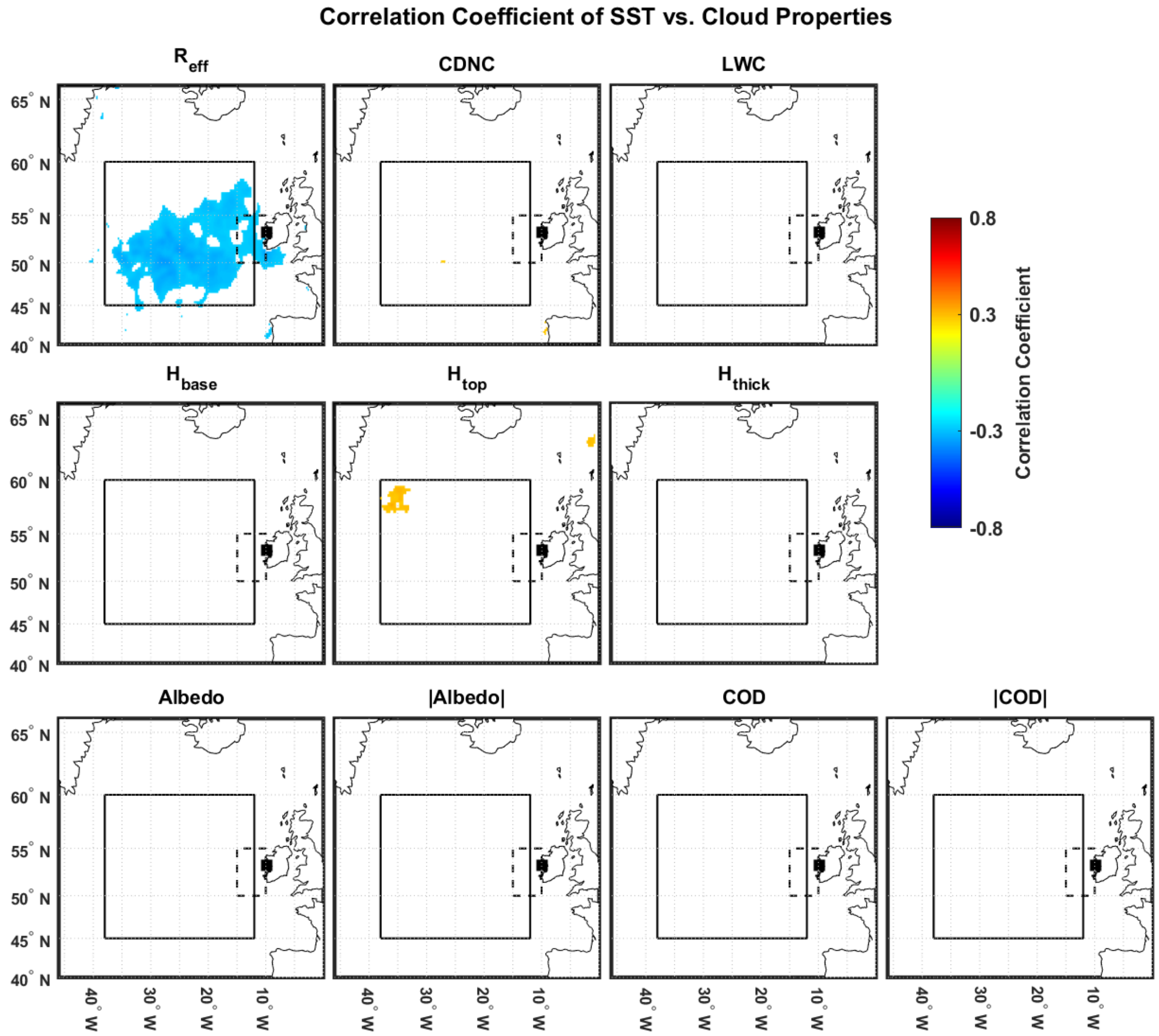

Figure S13. Same as Figure S12 but for sea surface temperature.

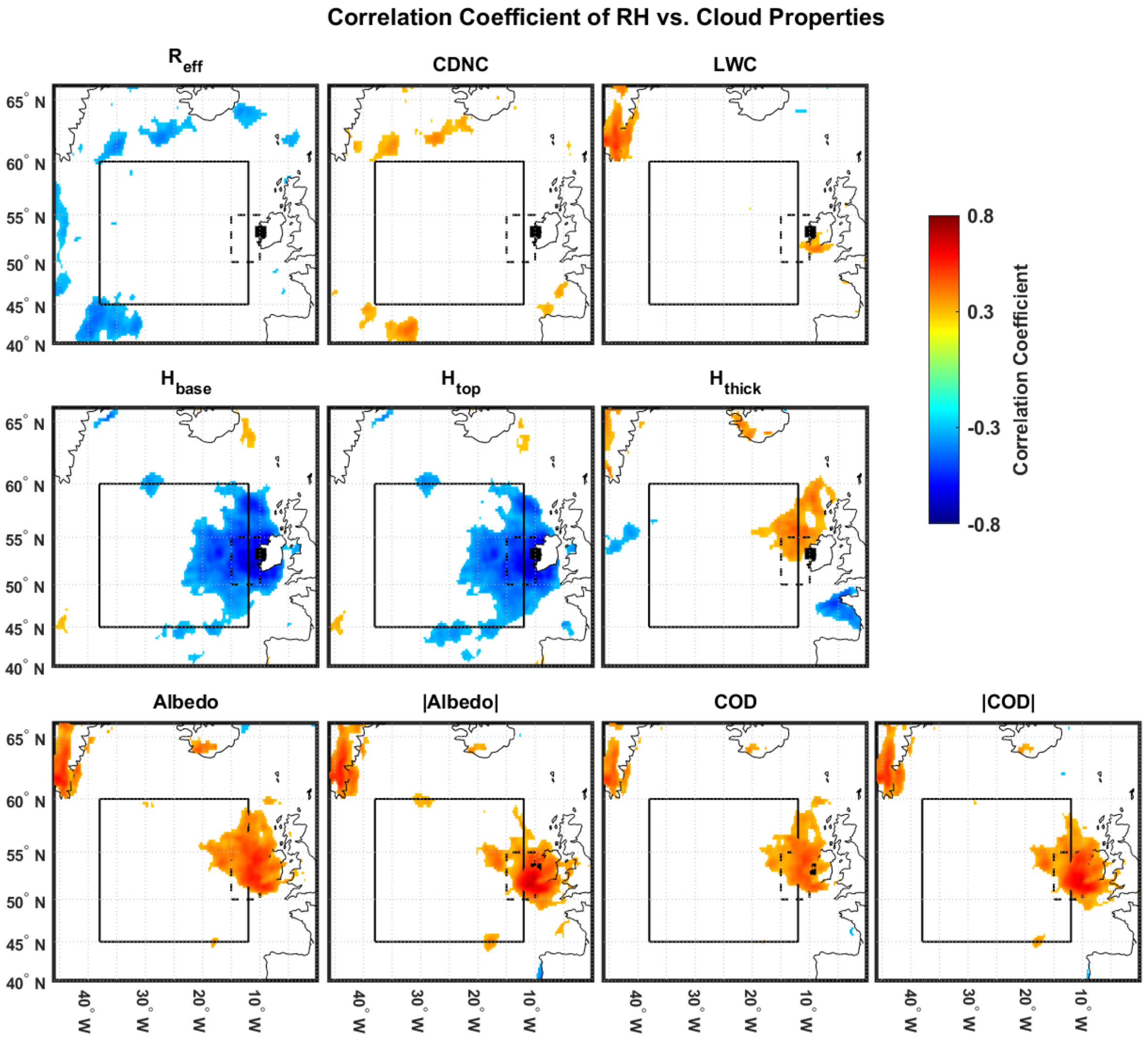

Figure S14. Same as Figure S12 but for relative humidity.

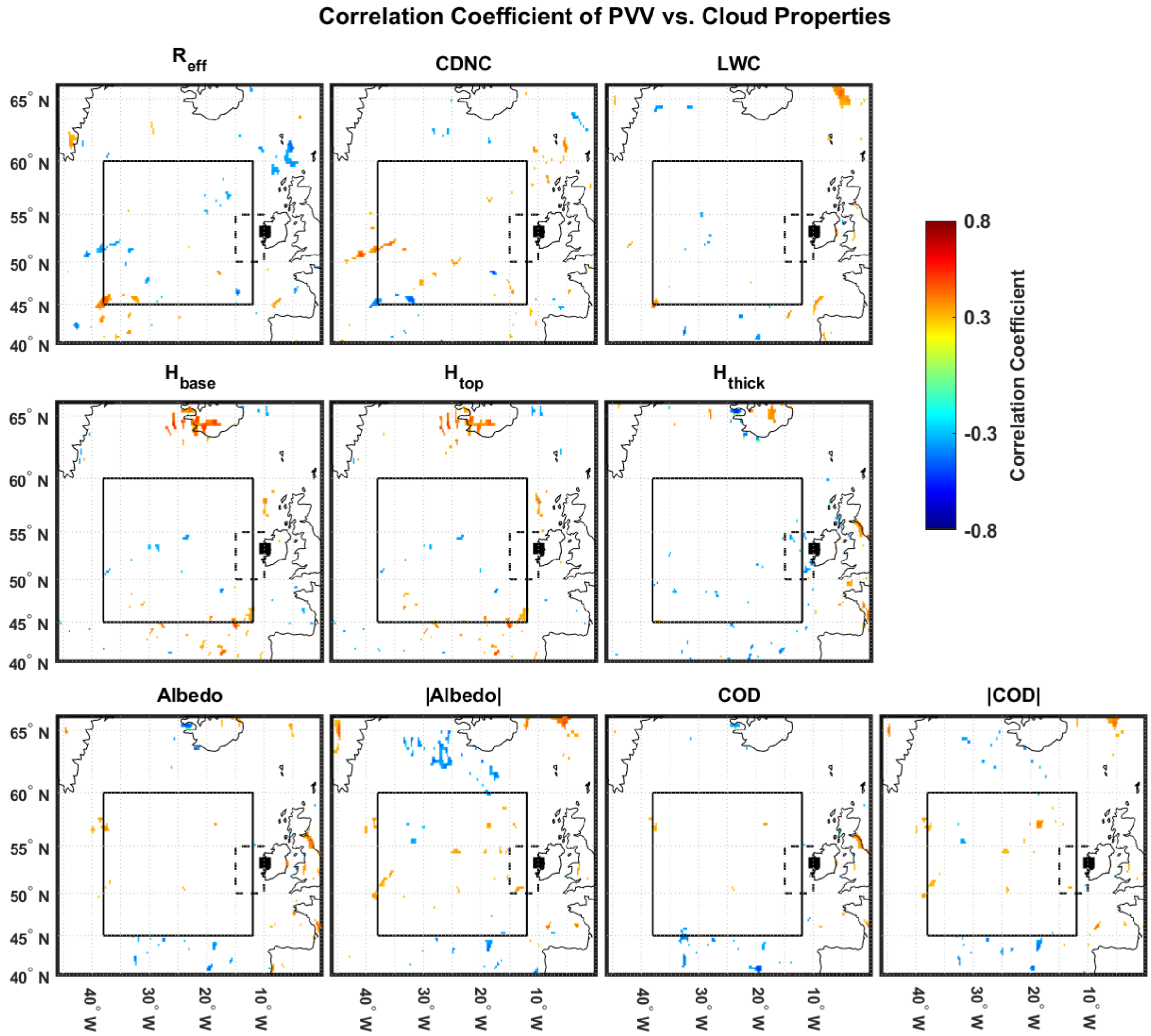

Figure S15. Same as Figure S12 but for pressure vertical velocity.

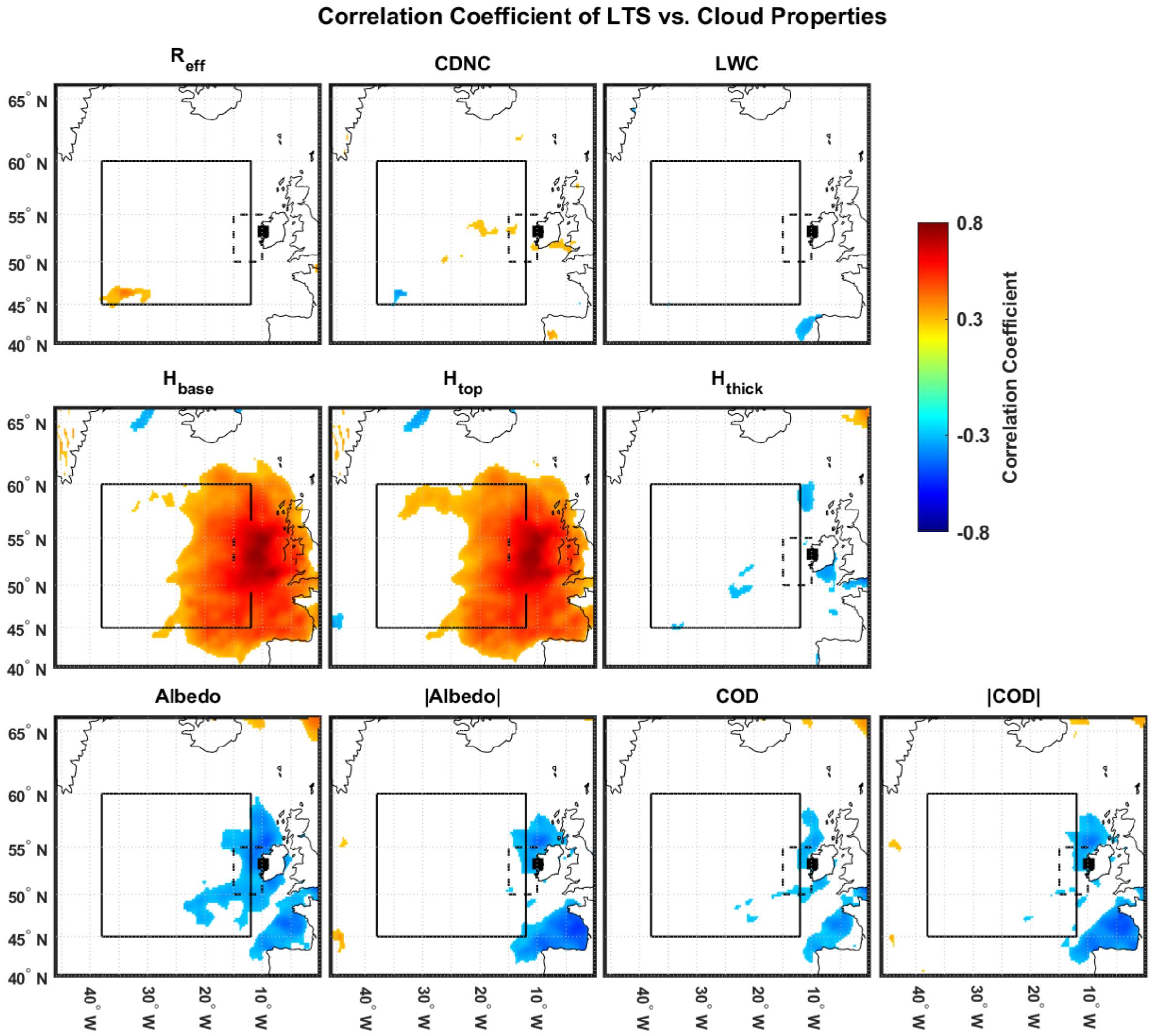

Figure S16. Same as Figure S12 but for low tropospheric stability.

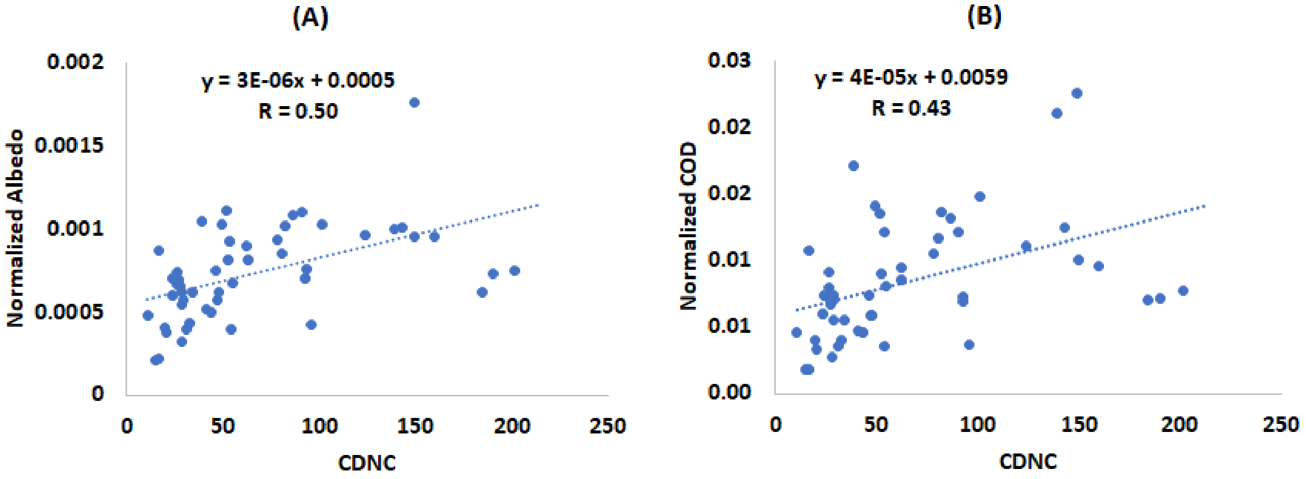

Figure S17. Scatter plot between (A) CDNC and normalized Albedo and (B) CDNC and normalized COD. The two linear fits are significant at a 95% confidence level.

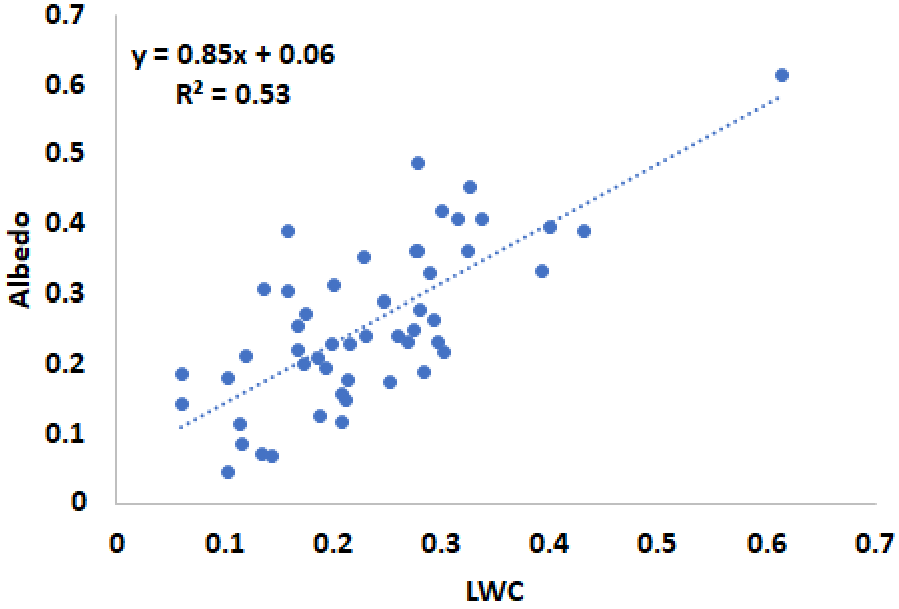

Figure S18. Scatter plot between LWC and Albedo. The linear fit is significant at a 95% confidence level.

	**Winter**	**Spring**	**Summer**	**Autumn**
Variable	**Mean±Std Dev**<br>**[Median]**<br>**(Min – Max)**			
CHL [mg m^{-3}]	0.21±0.04<br>[0.20]<br>(0.13 – 0.40)	0.35±0.13<br>[0.34]<br>(0.14 – 0.72)	0.49±0.09<br>[0.47]<br>(0.33 – 0.93)	0.31±0.08<br>[0.30]<br>(0.18 – 0.54)
R_{eff} [µm]	12.1±1.2<br>[12.5]<br>(9.6 – 13.2)	10.2±2.0<br>[11.1]<br>(7.1 – 12.7)	10.3±2.5<br>[10.8]<br>(5.4 – 14.2)	10.8±2.2<br>[11.1]<br>(7.2 – 14.5)
CDNC [cm^{-3}]	40.0±26.0<br>[28.5]<br>(16.8 – 86.3)	77.7±47.5<br>[56.8]<br>(16.4 – 159.8)	80.6±62.9<br>[54.2]<br>(14.9 – 213.3)	61.6±50.4<br>[41.4]<br>(10.9 – 149.4)
N_a [cm^{-3}]	105.8±61.6<br>[78.4]<br>(57.8 – 210.9)	248.2±52.0<br>[268.7]<br>(161.0 – 295.6)	326.2±186.5<br>[280.4]<br>(144.8 – 770.0)	140.5±61.1<br>[158.5]<br>(34.9 – 189.1)

Table S1. Seasonal statistics of CHL, R_{eff}, CDNC and N_a. The CHL statistics are obtained from daily mean data (2009-2015) within the outlined black box region in Figure 1 (Manuscript). CDNC and R_{eff} statistics are obtained from the considered 52 cloud cases. Most cases are representative of the summer season (24 cases), while 9 cases in both winter and autumn and 10 cases in spring. According to the nonparametric Mann-Whitney U-test, the differences between winter and summer are statistically significant at $p<0.05$ for CHL, R_{eff} and N_a and at $p<0.1$ for all the reported variables.

	Background Marine Conditions	Continental Polluted Cases
Variable	**Mean±Std Dev [Median] (Min – Max)**	
R_{eff} [µm]	10.7±2.2 [11.2] (5.4 – 14.5)	8.0±2.4 [8.3] (4.6 – 12.4)
CDNC [cm^{-3}]	69.7±54.0 [50.4] (10.9 – 213.3)	209.4±145.6 [160.2] (24.5 – 609.8)

Table S2. Statistical summary of R_{eff} and CDNC in background marine conditions vs. polluted cases.

Year	Month	Day	Start Hour	End Hour	Year	Month	Day	Start Hour	End Hour
2009	2	23	21	22	2011	6	19	8	8.5
2009	2	24	2	3	2011	6	30	4.5	5
2009	3	6	15.4	15.8	2011	7	25	22	22.5
2009	6	20	8	8.5	2011	7	26	1	1.5
2009	8	4	9.5	10	2011	8	2	1.5	2
2009	8	10	12	12.5	2011	8	2	17.7	18.3
2009	8	11	2.7	3	2011	8	21	4.25	4.6
2009	8	15	22.25	22.6	2011	8	21	8.1	8.3
2009	9	5	9.5	10	2011	8	22	5.5	6
2009	9	5	17.1	17.5	2011	9	14	15.2	15.7
2009	9	6	18.5	19	2011	10	12	10.5	10.8
2009	10	1	10	10.5	2012	2	14	23.1	23.4
2009	10	7	14	14.5	2012	3	10	5.7	6
2010	1	28	7	7.5	2012	3	11	11	11.4
2010	4	25	20.5	21	2012	3	20	6	6.5
2010	5	16	11.6	11.9	2013	8	9	19.8	20.1
2010	5	16	18	18.5	2013	8	21	14.5	15
2010	6	14	3	3.5	2013	9	3	5	6
2010	8	3	7.4	7.9	2014	5	8	12.1	12.4
2010	8	5	5.2	5.6	2014	6	19	12.5	12.7
2010	8	9	7.2	7.6	2014	6	20	14	14.5
2010	12	9	13.4	13.7	2014	7	3	16.6	17
2010	12	10	13.8	14.5	2014	8	15	22.9	23.3
2010	12	10	15	15.5	2014	11	30	21.5	22
2011	5	14	2.4	2.9	2014	12	20	17.7	18.2
2011	5	29	9	9.5	2015	1	27	11.1	11.4

Data Set S1. Measurement time of the selected 52 marine background cases.
